# Transcriptome-Wide Assessment of Human Brain and Lymphocyte Senescence

**DOI:** 10.1371/journal.pone.0003024

**Published:** 2008-08-20

**Authors:** Mun-Gwan Hong, Amanda J. Myers, Patrik K. E. Magnusson, Jonathan A. Prince

**Affiliations:** 1 Department of Medical Epidemiology and Biostatistics, Karolinska Institutet, Stockholm, Sweden; 2 Department of Psychiatry, University of Miami, Miller School of Medicine, Miami, Florida, United States of America; University of the Western Cape, South Africa

## Abstract

**Background:**

Identifying biological pathways that vary across the age spectrum can provide insight into fundamental mechanisms that impact disease and frailty in the elderly. Few methodological approaches offer the means to explore this question on as broad a scale as gene expression profiling. Here, we have evaluated mRNA expression profiles as a function of age in two populations; one consisting of 191 individuals with ages-at-death ranging from 65–100 years and with post-mortem brain mRNA measurements of 13,216 genes and a second with 1240 individuals ages 15–94 and lymphocyte mRNA estimates for 18,519 genes.

**Principal Findings:**

Among negatively correlated transcripts, an enrichment of mitochondrial genes was evident in both populations, providing a replication of previous studies indicating this as a common signature of aging. Sample differences were prominent, the most significant being a decrease in expression of genes involved in translation in lymphocytes and an increase in genes involved in transcription in brain, suggesting that apart from energy metabolism other basic cell processes are affected by age but in a tissue-specific manner. In assessing genomic architecture, intron/exon sequence length ratios were larger among negatively regulated genes in both samples, suggesting that a decrease in the expression of non-compact genes may also be a general effect of aging. Variance in gene expression itself has been theorized to change with age due to accumulation of somatic mutations and/or increasingly heterogeneous environmental exposures, but we found no evidence for such a trend here.

**Significance:**

Results affirm that deteriorating mitochondrial gene expression is a common theme in senescence, but also highlight novel pathways and features of gene architecture that may be important for understanding the molecular consequences of aging.

## Introduction

A decline in cell function with advancing age is a ubiquitous characteristic of all organisms. In humans, the effects of aging become manifest on a variety of levels that extend from an accumulation of DNA mutations to lipid oxidation, protein modification, cell loss, and ultimately death that is primarily due to increased susceptibility to age-related diseases [1]. Apart from overt changes, such as declining muscle strength, extensive metabolic alterations also occur with aging, one of the most prominent being impaired glucose tolerance [2]. Two central evolutionary theories hypothesize that the detrimental effects of aging are due to an accumulation of mutations, or antagonistic pleiotropy, whereby genes with beneficial effects early in life become deleterious with age [3]. These are not necessarily exclusive, and there is at present relatively strong evidence for both in studies of model organisms and in natural populations [3], [4].

Whatever the cause, there is value in charting the molecular sequelae of aging on as broad a scale as possible. Few other methodological approaches lend themselves as well to this as mRNA expression profiling. There have been a handful of studies that have attempted to catalogue how mRNA expression changes with age, the largest of which have been performed in kidney [5] and muscle samples [6]. An intriguing conclusion from the latter study is that there may be a common set of genes that change equivalently in different tissues. For example genes that make up the mitochondrial electron transport chain appear to decrease with age in different tissues, and this is supported in that decreases are also evident in mice and flies [6]. Importantly however, these studies remain relatively small in scale and few in number thus meriting larger studies in additional populations and tissues.

The effects of aging are particularly pronounced in the human brain where characteristic changes in morphology include a reduction in both neuronal size and synaptic density [7], [8]. On a behavioral level, decreases in motor and cognitive function are hallmarks of normal aging [9]. Dementia is the most prevalent disorder of the human brain, affecting 20–25 million people worldwide and representing a tremendous burden in terms of years of suffering with disability [10]. To date, there has been only one study, involving 30 individuals, on the effects of aging on gene expression at a transcriptome-level in the normal human brain [11]. There the authors described a number of pathways affected by aging and also concluded that genes for which down-regulation was evident had an over-representation of mutation in gene promoters. A recent study examined the effects of gene polymorphism on gene expression in a relatively large set of human brain samples [12]. An attractive feature of that study was that individuals, prior to death, were free of neurological disease. Our primary focus in the present study has been to use the above sample to investigate the question of whether general changes in gene expression occur in the aging human brain. This has been complemented with an analysis of lymphocyte mRNA expression in order to explore for common molecular themes in different tissues as well as to enable an assessment of a broader age range than that typically available in post-mortem samples.

## Results

We began by performing several validation steps towards the initial goal of evaluating if age-related change in gene expression could be detected in the human brain. First, evidence was sought for systematic outlier effects among the 2,096,975 individual expression level estimates. We identified all expression values that were more than 3.4644 standard deviations from the mean expression value for that particular transcript (see methods for an explanation for choosing this threshold). With this, we expected to see approximately 1,086 outliers by chance in the entire set assuming normal distributions of the log_10_ transformed data. There were 7,079 outliers identified in this way, and these were eliminated from all further analyses. Second, the focus of the original study was primarily upon brain cortex, with 5 subsets being represented, 3 cortical, one group of 6 cerebellum samples and an additional group of samples for which no specific region could be assigned. We assessed whether these classifications differed systematically with regards to transcript detection rates and global expression levels using ANOVA (see methods). Across the various brain region categories there were no significant differences for any these covariates. Simple linear regression was also performed for age-at-death and pmi versus global expression level. For this, there was marginal evidence that age-at-death correlated with global expression (P = 0.080, r^2^ = 0.016). For pmi however, the trend was significant, but the direction of the curve suggested that expression was increasing with increasing pmi (P = 0.0039. r^2^ = 0.046). Of note, linear regression of the 2 global expression covariates (see methods) and detection rate metrics (one reflecting all 24,357 transcripts probed by the Illumina HumanRefseq-8 chip and the other reflecting the 14,078 transcripts detected in this brain sample) all showed strong correlation (r^2^ in excess of 0.5 for all 6 comparisons). Based upon all of the above tests, we resolved to use the global average expression level that includes all 14,078 transcripts as a primary metric of quality as a covariate for initial analyses.

**Figure 1:**
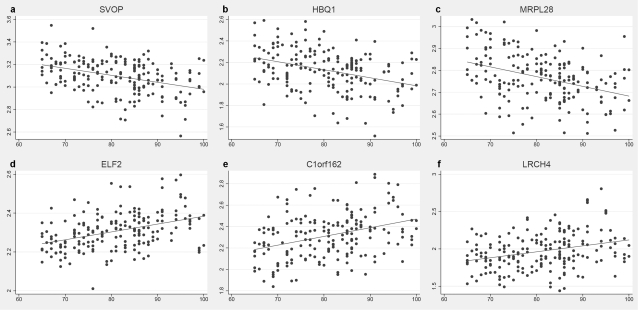
Scatter plots and fitted linear regression lines of the most significantly associated down-regulated (a–c) and up-regulated (d–f) genes with age-at-death in brain samples.

For the brain sample, we regressed age-at-death on transcript levels one at a time for all 14,078 transcripts that had been detected in 5 or more individuals in the original study. The results of this are shown in Tables 1 and 2, where we document 54 transcripts that exhibited significant change after Bonferroni correction for 14,078 tests. For purposes of illustration, regression lines for the top 6 scoring genes (3 up-regulated and 3 down-regulated) are also displayed in Figure 1. For this analysis, the most significant finding was for the *SVOP* gene (encoding a synaptic vesicle protein), and the single strongest effect in terms of variance explained was for the *TAC3* gene (encoding tachykinin 3). The smallest number of individuals for which a significant correlation with age was detected was 133 (also for the *TAC3* gene). We noted that 3 of the top scoring 54 genes were also present in a list of 532 putative “housekeeping” genes (see [13]), these being *PIN1*, *GUK1*, and *ARPC2*. This proportion is not significantly different from what would be expected by chance (3 vs. 51 compared to 532 vs. 6968). At this stage, we also scrutinized this list of 54 genes more closely for the potential impact of covariates. For this, multiple regression models were fitted as above, but for each transcript in turn we also included terms for pmi, brain region, or gender. All 54 genes remained highly significant when any of these were individually tested (not shown). Among the top 54 transcripts, all were detectable in more than 100 individuals, reinforcing the importance of power in analyses such as this. There were however a few cases where a small number of observations gave rise to apparently large effects. The gene that ranked as the 139^th^ most significant finding (falling below the multiple test correction threshold), *ZIC3*, had only 14 expression level observations and an r^2^ = 0.76 (P = 2.6×10^−5^). This highlighted the possibility that genes with lower maximum expression levels might be changing to a larger degree with age, but technologies with lower detection thresholds and/or larger sample sizes would be required to identify them.

**Table 1:** Genes whose expression decreases with age in the human brain.

Symbol	Description	Chr.Pos	*P* value^a^	R^2 b^
SVOP	SV2 related protein homolog (rat)	12q	4.3(10^−9^)	0.14
HBQ1	hemoglobin, theta 1	16p	8.6(10^−8^)	0.13
MRPL28	mitochondrial ribosomal protein L28	16p	1.0(10^−7^)	0.14
TAC3	tachykinin 3 (neuromedin K, neurokinin beta)	12q	1.0(10^−7^)	0.18
C6orf154	chromosome 6 open reading frame 154	6p	1.5(10^−7^)	0.13
SLC25A6	solute carrier family 25, member A6	Xp/Yp	1.9(10^−7^)	0.14
ST3GAL2	ST3 beta-galactoside alpha-2,3-sialyltransferase 2	16q	2.2(10^−7^)	0.15
PIN1	peptidylprolyl cis/trans isomerase, NIMA-interacting 1	19p	3.2(10^−7^)	0.13
ARPC2	actin related protein 2/3 complex, subunit 2, 34 kDa	2q	3.2(10^−7^)	0.14
KCNF1	potassium voltage-gated channel, subfamily F, member 1	2p	4.1(10^−7^)	0.12
GSS	glutathione synthetase	20q	4.3(10^−7^)	0.11
LOC255849	hypothetical LOC255849		4.5(10^−7^)	0.12
HMGB3	high-mobility group box 3	Xq	4.8(10^−7^)	0.13
TMEM121	transmembrane protein 121	14q	6.1(10^−7^)	0.14
CAMK2N1	calcium/calmodulin-dependent protein kinase II inhibitor 1	1p	6.4(10^−7^)	0.12
OLFM1	olfactomedin 1	9q	7.4(10^−7^)	0.13
KCNIP1	Kv channel interacting protein 1	5q	9.2(10^−7^)	0.12
FABP3	fatty acid binding protein 3	1p	1.1(10^−6^)	0.08
GUK1	guanylate kinase 1	1q	1.2(10^−6^)	0.13
COPS7A	COP9 constitutive photomorphogenic homolog subunit 7A (Arabidopsis)	12p	1.3(10^−6^)	0.07
VIP	vasoactive intestinal peptide	6q	1.4(10^−6^)	0.11
PQLC1	PQ loop repeat containing 1	18q	1.6(10^−6^)	0.13
FLJ34048	hypothetical transcript		1.6(10^−6^)	0.12
CYP46A1	cytochrome P450, family 46, subfamily A, polypeptide 1	14q	2.0(10^−6^)	0.11
ATG7	ATG7 autophagy related 7 homolog (S. cerevisiae)	3p	2.1(10^−6^)	0.11
CXCL14	chemokine (C-X-C motif) ligand 14	5q	2.3(10^−6^)	0.13
NXPH1	neurexophilin 1	7p	2.6(10^−6^)	0.11
C17orf76	chromosome 17 open reading frame 76		2.7(10^−6^)	0.17
NPM3	nucleophosmin/nucleoplasmin, 3	10q	3.1(10^−6^)	0.08
LHX6	LIM homeobox 6	9q	3.2(10^−6^)	0.07
FRMPD2	FERM and PDZ domain containing 2	10q	3.4(10^−6^)	0.07
HSD11B1L	hydroxysteroid (11-beta) dehydrogenase 1-like	19p	3.5(10^−6^)	0.12
SMAD3	SMAD family member 3	15q	3.5(10^−6^)	0.09

a *P*-value with the null hypothesis *β*_1_ = 0 in linear regression model *Y* = *β*_0_+*β*_1_ *x*_1_+*β*_2_ *x*_2_+*ε*, where *Y* is transcript expression, *x*_1_ age-at-death, *x*_2_ global expression, and *ε* random error. ^b^Coefficient of determination of linear regression model *Y* = *β*_0_+*β*_1_ *x*_1_+*ε*, where each variable is as described above.

**Table 2:** Genes whose expression increases with age in the human brain.

Symbol	Description	Chr.Pos	*P* value^a^	R^2 b^
ELF2	E74-like factor 2 (ets domain transcription factor)	4q	5.3(10^−9^)	0.16
C1orf162	chromosome 1 open reading frame 162	1p	6.0(10^−9^)	0.13
LRCH4	leucine-rich repeats and calponin homology (CH) domain containing 4	7q	2.9(10^−8^)	0.09
MTUS1	mitochondrial tumor suppressor 1	8p	6.8(10^−8^)	0.12
RUFY1	RUN and FYVE domain containing 1	5q	7.0(10^−8^)	0.10
RDH5	retinol dehydrogenase 5 (11-cis/9-cis)	12q	1.4(10^−7^)	0.06
TYK2	tyrosine kinase 2	19p	1.8(10^−7^)	0.10
CLK1	CDC-like kinase 1	2q	2.1(10^−7^)	0.14
TXNIP	thioredoxin interacting protein	1q	2.1(10^−7^)	0.10
SLC16A9	solute carrier family 16 (monocarboxylic acid transporters), member 9	10q	4.8(10^−7^)	0.06
ADORA3	adenosine A3 receptor	1p	5.1(10^−7^)	0.10
UCKL1	uridine-cytidine kinase 1-like 1	20q	7.4(10^−7^)	0.11
CTDSP2	nuclear LIM interactor-interacting factor 2	12q	1.1(10^−6^)	0.05
HLA-DPB1	major histocompatibility complex, class II, DP beta 1	6p	1.2(10^−6^)	0.09
PATL1	protein associated with topoisomerase II homolog 1 (yeast)	11q	1.4(10^−6^)	0.12
GDPD3	glycerophosphodiester phosphodiesterase domain containing 3	16p	1.4(10^−6^)	0.07
BHLHB3	basic helix-loop-helix domain containing, class B, 3	12p	1.7(10^−6^)	0.07
RNASE4	ribonuclease, RNase A family, 4	14q	1.9(10^−6^)	0.06
PLEKHM1	pleckstrin homology domain containing, family M (with RUN domain) member 1	17q	2.5(10^−6^)	0.04
FAM46A	family with sequence similarity 46, member A	6q	3.4(10^−6^)	0.06
CALCOCO1	calcium binding and coiled-coil domain 1	12q	3.5(10^−6^)	0.09

a *P*-value with the null hypothesis *β*_1_ = 0 in linear regression model *Y* = *β*_0_+*β*_1_ *x*_1_+*β*_2_ *x*_2_+*ε*, where *Y* is transcript expression, *x*_1_ age-at-death, *x*_2_ global expression, and *ε* random error. ^b^Coefficient of determination of linear regression model *Y* = *β*_0_+*β*_1_ *x*_1_+*ε*, where each variable is as described above.

We contrasted the individual transcript results of this analysis with the first study to address the impact of aging on expression at the transcriptome level [11]. In that study, 30 individuals were included with an age range of 26–106. Our set was scrutinized for the 148 genes previously highlighted to be associated with aging (see specifically table 1 from [11]). Only one gene from our set that survived strict multiple testing correction was also present in their set, this being *MRPL28*. With a relaxed uncorrected significance threshold in our set of 0.005, we found 26 overlapping genes. This proportion is significant given that we observed 1,141 transcripts in our set that were significant at the 0.005 threshold, suggesting that some replication exists, albeit limited (26 vs. 122 compared to 1,141 vs. 12,937). In our set, the majority of significant genes exhibited a decrease in expression with advancing age. An intriguing aspect of this comparison was that among the 26 overlapping genes, there were 9 for which expression increased with age in the original study, and for all 9 these were increased with age in the present study. Due to the consistency in the direction of the effects, this might be considered a stronger case for replication. Seven of these 9 genes were previously considered as a class of stress response genes.

We explored an expanded list of genes that exceeded a relaxed significance threshold (uncorrected P<0.05) for enrichment or deficit based upon Gene Ontology (GO) terms and the Kyoto Encyclopedia of Genes and Genomes (KEGG) pathways. The entire set of genes was divided into two groups based upon whether expression was increasing or decreasing with age. We then searched for over- or under-represented terms and pathways in each list of genes using the DAVID web application [14]. For this we created a diminished non-redundant set of 13,216 base genes from the total set of 14,078 by eliminating multiple transcripts representing the same gene (see methods). The enriched terms (Bonferroni corrected *P*<0.05) are shown in Tables 3 and 4, excluding the ancestor terms that became significant mainly by an overrepresented descendant in the GO structure. A larger list of all overrepresented terms and pathways with a less stringent threshold (uncorrected *P*<0.01) is shown in Tables S1 and S2. In summarizing these results, the genes whose products are involved in DNA-binding were the most significantly overrepresented group among genes whose expression increases with age (Table 4). Genes with products involved in the regulation of DNA-dependent transcription and genes encoding proteins located in or a subcomponent of the nucleus were also significantly enriched. Among all genes negatively correlated with age, the most significantly enriched groups included genes involved in nervous system development, mitochondrial genes, and those whose products are constituents of the cytoplasm (Table 3). We also highlighted the degree of enrichment in terms of a fold change (the number of genes that give rise to this number are shown in Tables S1 and S2). Thus, in quantitative terms the most enriched genes occurred in the set of negatively correlated transcripts, where dendrite genes and genes associated with neuronal projections were enriched more than 3 fold (Table 3). In contrast, although highly significant in some cases, genes whose expression is increasing with age are enriched a maximum of 1.80 fold (Table 4). As a comparison, the probabilities for the enriched terms to be underrepresented by chance within the opposite group of genes are also shown in the last two columns of Tables 3 and 4. For example, from Table 4, we show that the term “nucleus” which is significantly enriched in the set of genes that are increasing with age is also in deficit in the set of genes that are decreasing with age.

**Table 3:** Terms in the Gene Ontology and KEGG pathway databases over-represented among genes that decreased expression with advancing age in the human brain (1,450 genes in total 13,216).

Category	Term	*P* value^a^	Bonferroni	Fold^b^	Increasing group^c^	
					Fold^b^	*P* value^d^
GO Bio.Process	nervous system development	7.8(10^−9^)	3.8(10^−5^)	1.73	0.76	6.7(10^−3^)
	synaptic transmission	7.4(10^−7^)	3.5(10^−3^)	2.07	0.28	3.1(10^−7^)
	oxidative phosphorylation	1.4(10^−6^)	6.7(10^−3^)	2.73	0.15	5.3(10^−5^)
GO Cell.Component	cytoplasmic part	9.0(10^−11^)	7.2(10^−8^)	1.29	0.76	4.2(10^−11^)
	mitochondrion	1.5(10^−10^)	1.2(10^−7^)	1.65	0.54	7.7(10^−10^)
	neuron projection	2.3(10^−10^)	1.8(10^−7^)	3.41	0.40	1.0(10^−2^)
	synapse	1.8(10^−9^)	1.4(10^−6^)	2.55	0.42	6.4(10^−4^)
	mitochondrial membrane part	8.7(10^−9^)	6.9(10^−6^)	2.95	0.14	1.8(10^−5^)
	mitochondrial inner membrane	3.1(10^−8^)	2.5(10^−5^)	2.11	0.45	5.4(10^−5^)
	mitochondrial respiratory chain	4.9(10^−6^)	3.9(10^−3^)	2.99	0.11	4.3(10^−4^)
	dendrite	5.2(10^−6^)	4.2(10^−3^)	3.60	0.17	1.1(10^−2^)
GO Mol.Function	hydrogen ion transmembrane transporter activity	1.8(10^−6^)	4.7(10^−3^)	2.70	0.58	5.1(10^−2^)
KEGG Pathway	Oxidative phosphorylation	1.9(10^−9^)	3.8(10^−7^)	2.66	0.25	1.4(10^−4^)

a EASE-score, *P-*value of a modified Fisher's exact test for overrepresentation [35]. ^b^Fold enrichment in each gene group compared to the base set. ^c^Gene group with expression that increases with advancing age-at-death (same genes used in Table 4, 1943 genes). ^d^Hypergeometric test for underrepresentation using annotated genes from a total set of 13,216 genes as base population [36].

**Table 4:** Terms in the Gene Ontology and KEGG pathway databases over-represented among genes that increased expression with advancing age in the human brain (1,943 genes in total 13,216).

Category	Term	*P* value^a^	Bonferroni	Fold^b^	Decreasing group^c^	
					Fold^b^	*P* value^d^
GO Bio.Process	regulation of transcription, DNA-dependent	2.1(10^−13^)	1.0(10^−9^)	1.43	0.56	6.1(10^−13^)
	chromosome organization and biogenesis	1.4(10^−7^)	6.8(10^−4^)	1.80	0.53	1.4(10^−3^)
	DNA packaging	4.7(10^−6^)	2.2(10^−2^)	1.78	0.49	1.9(10^−3^)
	DNA metabolic process	6.4(10^−6^)	3.0(10^−2^)	1.45	0.59	7.6(10^−5^)
GO Cel.Component	nucleus	1.2(10^−13^)	9.6(10^−11^)	1.26	0.75	3.7(10^−11^)
GO Mol.Function	DNA binding	6.8(10^−14^)	1.7(10^−10^)	1.44	0.52	1.1(10^−14^)
	zinc ion binding	1.7(10^−11^)	4.3(10^−8^)	1.39	0.64	6.3(10^−9^)
	transcription regulator activity	1.1(10^−6^)	2.7(10^−3^)	1.38	0.65	1.8(10^−5^)

a EASE-score, *P-*value of a modified Fisher's exact test for overrepresentation [35]. ^b^Fold enrichment in each gene group compared to the base set. ^c^Gene group with expression that decreases with advancing age-at-death (same genes used in Table 3, 1450 genes). ^d^Hypergeometric test for underrepresentation using annotated genes from a total set of 13,216 genes as base population [36].

**Figure 2:**
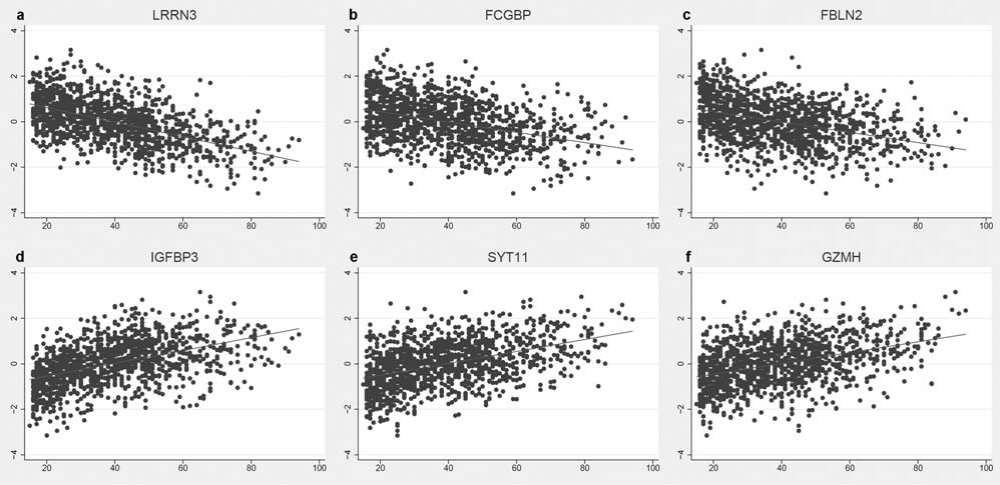
Scatter plots and fitted linear regression lines of the most significantly associated down-regulated (a–c) and up-regulated (d–f) genes with age in lymphocyte samples.

We extended the above analytical scheme to a second sample consisting of 1240 individuals with lymphocyte mRNA measures for 19,648 transcripts representing 18,519 genes. We note that a rigorous standardization procedure had been applied to this set previously [15] and we thus elected not to perform any additional transformations, nor to include any additional covariates (see methods). Linear regression was performed as above on all 19,648 transcripts versus age for this sample. Given its size, and compared to the brain sample, there were many more transcripts that exceeded a strict Bonferroni significance threshold, and we chose to present only the top 50 (25 negatively and 25 positively correlated with age) which are shown in Tables 5 and 6, arbitrarily truncated from a total of 1080 (612 negative and 468 positive). The top 3 scoring negatively regulated genes and top 3 scoring positively regulated genes are shown in Figure 2. The most significant finding in this set was for the *LRRN3* (encoding a membrane protein with unknown function), and this was also the gene upon which age exhibited the largest effect (28% variance explained). Interestingly, the second largest effect observed in this set was 14% variance explained, representing in our view an anomalous drop from the top ranking gene. As was done for the brain sample, we again divided this list into up- and down- regulated genes and performed term/pathway based analyses. An arbitrary significance level of 0.01 was chosen from the linear regression results to establish these 2 lists (this differed from the brain sample for which a threshold of 0.05 was set due to the number of genes exceeding significance in lymphocytes and a 2000 gene limit set by the DAVID web tool). The total set of 19,648 transcripts for which regression was performed, was reduced to 13,231 for which annotations could be obtained towards analysis using the DAVID web application. The results of this are shown in Tables 7 and 8 where Bonferroni corrected (P<0.05) enriched terms and pathways are documented. As was done for the brain sample, an expanded list of enriched terms and pathways according to relaxed uncorrected significance threshold of P<0.01 is shown in Tables S3 and S4. The main highlight in our view is the highly significant enrichment of mitochondrial genes in the negatively regulated group (Table 7), providing a replication of what was seen in the brain. In general, the magnitude of the fold-change in term/pathway enrichment was larger in the set of negatively regulated genes, also in agreement with what was seen in brain.

**Table 5:** Genes whose expression decreases with age in human lymphocytes.

Symbol	Description	Chr.Pos	*P* value^a^	R^2^
LRRN3	leucine rich repeat neuronal 3	7q	5.1(10^−94^)	0.29
FCGBP	Fc fragment of IgG binding protein	19q	7.5(10^−44^)	0.14
FBLN2	fibulin 2	3p	8.1(10^−44^)	0.14
NRCAM	neuronal cell adhesion molecule	7q	3.3(10^−43^)	0.14
ITM2C	integral membrane protein 2C	2q	1.8(10^−39^)	0.13
PDE9A	phosphodiesterase 9A	21q	4.9(10^−37^)	0.12
ZNF154	zinc finger protein 154	19q	5.3(10^−36^)	0.12
ZSCAN18	zinc finger and SCAN domain containing 18	19q	4.7(10^−35^)	0.12
SATB1	SATB homeobox 1	3p	9.8(10^−35^)	0.11
FLNB	filamin B, beta (actin binding protein 278)	3p	1.1(10^−34^)	0.11
FAM134B	family with sequence similarity 134, member B	5p	3.1(10^−34^)	0.11
SCD	stearoyl-CoA desaturase (delta-9-desaturase)	10q	8.8(10^−34^)	0.11
SREBF1	sterol regulatory element binding transcription factor 1	17p	4.5(10^−33^)	0.11
CCR7	chemokine (C-C motif) receptor 7	17q	6.3(10^−33^)	0.11
PHGDH	phosphoglycerate dehydrogenase	1p	1.1(10^−32^)	0.11
LEF1	lymphoid enhancer-binding factor 1	4q	5.3(10^−32^)	0.11
NPM3	nucleophosmin/nucleoplasmin, 3	10q	9.8(10^−32^)	0.11
OXNAD1	oxidoreductase NAD-binding domain containing 1	3p	2.1(10^−31^)	0.10
TNNT3	troponin T type 3 (skeletal, fast)	11p	3.4(10^−30^)	0.10
PLEKHG4		16q	5.8(10^−30^)	0.10
MGC9913	hypothetical protein MGC9913		1.5(10^−29^)	0.10
SLC7A6	solute carrier family 7 (cationic amino acid transporter, y+ system), member 6	16q	4.4(10^−29^)	0.10
CD27	CD27 molecule	12p	6.1(10^−29^)	0.10
AEBP1	AE binding protein 1	7p	1.0(10^−28^)	0.10
MGC29506	hypothetical protein MGC29506	5q	1.2(10^−28^)	0.09

a *P*-value with the null hypothesis *β*_1_ = 0 in linear regression model *Y* = *β*_0_+*β*_1_ *x*_1_+*ε*, where *Y* is transcript expression, *x*_1_ age, and *ε* random error.

**Table 6:** Genes whose expression increases with age in human lymphocytes.

Symbol	Description	Chr.Pos	*P* value^a^	R^2^
IGFBP3	insulin-like growth factor binding protein 3	7p	1.7(10^−70^)	0.22
SYT11	synaptotagmin XI	1q	7.4(10^−60^)	0.19
GZMH	granzyme H (cathepsin G-like 2, protein h-CCPX)	14q	2.0(10^−48^)	0.16
JAKMIP1	janus kinase and microtubule interacting protein 1	4p	5.4(10^−45^)	0.15
RCAN2	regulator of calcineurin 2	6p	1.5(10^−43^)	0.14
CRIP1	cysteine-rich protein 1 (intestinal)	14q	1.9(10^−39^)	0.13
PATL2	protein associated with topoisomerase II homolog 2 (yeast)		1.2(10^−38^)	0.13
MSC	musculin (activated B-cell factor-1)	8q	5.9(10^−32^)	0.11
GDPD5	glycerophosphodiester phosphodiesterase domain containing 5	11q	3.1(10^−30^)	0.10
APOBEC3H	apolipoprotein B mRNA editing enzyme, catalytic polypeptide-like 3H	22q	2.3(10^−29^)	0.10
CCL5	chemokine (C-C motif) ligand 5	17q	7.1(10^−29^)	0.10
GFI1	growth factor independent 1 transcription repressor	1p	2.4(10^−28^)	0.09
MANEAL	mannosidase, endo-alpha-like	1p	2.9(10^−28^)	0.09
KIF21A	kinesin family member 21A	12q	3.1(10^−27^)	0.09
GPR137B	G protein-coupled receptor 137B	1q	5.4(10^−27^)	0.09
PDGFRB	platelet-derived growth factor receptor, beta polypeptide	5q	3.7(10^−26^)	0.09
PCBP4	poly(rC) binding protein 4	3p	8.1(10^−26^)	0.09
B3GAT1	beta-1,3-glucuronyltransferase 1 (glucuronosyltransferase P)	11q	1.1(10^−25^)	0.08
LLGL2	lethal giant larvae homolog 2 (Drosophila)	17q	4.7(10^−25^)	0.08
LAG3	lymphocyte-activation gene 3	12p	5.1(10^−25^)	0.08
PPP2R2B	beta isoform of regulatory subunit B55, protein phosphatase 2 isoform b	5q	1.5(10^−24^)	0.08
	hypothetical gene supported by BC040060		9.1(10^−24^)	0.08
PRSS23	protease, serine, 23	11q	1.5(10^−23^)	0.08
B4GALT5	UDP-Gal:betaGlcNAc beta 1,4- galactosyltransferase, polypeptide 5	20q	1.9(10^−22^)	0.07
MXRA7	matrix-remodelling associated 7	17q	3.0(10^−22^)	0.07

a *P*-value with the null hypothesis *β*_1_ = 0 in linear regression model *Y* = *β*_0_+*β*_1_ *x*_1_+*ε*, where *Y* is transcript expression, *x*_1_ age, and *ε* random error.

**Table 7:** Terms in the Gene Ontology and KEGG pathway databases over-represented among genes that decreased expression with advancing age in human lymphocytes (1,878 genes in total 13,232).

Category	Term	*P* value^a^	Bonferroni	Fold^b^	Increasing group^c^	
					Fold^b^	*P* value^d^
GO Bio.Process	translation	4.2(10^−21^)	2.0(10^−17^)	2.19	0.60	8.7(10^−4^)
	cellular biosynthetic process	6.7(10^−21^)	3.2(10^−17^)	1.83	0.79	1.5(10^−2^)
	gene expression	4.0(10^−18^)	1.9(10^−14^)	1.36	0.70	3.1(10^−11^)
	ribosome biogenesis and assembly	1.5(10^−8^)	7.2(10^−5^)	2.90	0.23	5.9(10^−3^)
	tRNA metabolic process	2.9(10^−8^)	1.4(10^−4^)	2.57	0.52	4.7(10^−2^)
	RNA metabolic process	2.2(10^−7^)	1.0(10^−3^)	1.24	0.71	6.9(10^−9^)
	RNA processing	8.9(10^−7^)	4.2(10^−3^)	1.61	0.23	2.5(10^−11^)
	tRNA processing	4.3(10^−6^)	2.0(10^−2^)	2.82	0.63	2.2(10^−1^)
	rRNA processing	8.4(10^−6^)	3.9(10^−2^)	2.80	0.16	1.1(10^−2^)
GO Cell.Component	ribosome	2.0(10^−23^)	1.5(10^−20^)	3.02	0.09	1.1(10^−8^)
	ribosomal subunit	4.2(10^−21^)	3.3(10^−18^)	3.59	0.08	1.1(10^−5^)
	ribonucleoprotein complex	1.6(10^−20^)	1.3(10^−17^)	2.19	0.22	3.5(10^−12^)
	organelle lumen	9.0(10^−16^)	7.0(10^−13^)	1.71	0.55	2.2(10^−7^)
	mitochondrion	5.0(10^−12^)	3.9(10^−9^)	1.62	0.60	7.4(10^−6^)
	small ribosomal subunit	2.7(10^−11^)	2.2(10^−8^)	3.72	0.00	1.1(10^−3^)
	large ribosomal subunit	1.4(10^−10^)	1.1(10^−7^)	3.45	0.14	4.6(10^−3^)
	cytosolic part	3.1(10^−10^)	2.5(10^−7^)	2.60	0.63	8.0(10^−2^)
	nucleolus	5.6(10^−10^)	4.4(10^−7^)	2.42	0.47	8.7(10^−3^)
	intracellular organelle part	4.9(10^−9^)	3.8(10^−6^)	1.24	0.83	1.4(10^−4^)
	mitochondrial part	6.4(10^−9^)	5.0(10^−6^)	1.72	0.63	1.9(10^−3^)
	mitochondrial matrix	1.5(10^−8^)	1.2(10^−5^)	2.25	0.39	1.8(10^−3^)
	mitochondrial small ribosomal subunit	4.5(10^−5^)	3.4(10^−2^)	4.25	0.00	1.1(10^−1^)
GO Mol.Function	structural constituent of ribosome	9.4(10^−22^)	2.4(10^−18^)	3.08	0.10	1.2(10^−7^)
	nucleic acid binding	2.4(10^−19^)	6.0(10^−16^)	1.40	0.64	1.1(10^−14^)
	RNA binding	2.6(10^−19^)	6.7(10^−16^)	1.95	0.34	1.3(10^−11^)
	methyltransferase activity	3.0(10^−6^)	7.6(10^−3^)	2.11	0.33	1.3(10^−3^)
	oxidoreductase activity (NAD or NADP)	5.2(10^−6^)	1.3(10^−2^)	2.60	0.76	3.1(10^−1^)
KEGG Pathway	Ribosome	1.1(10^−17^)	2.2(10^−15^)	3.97	0.00	3.4(10^−5^)

a EASE-score, *P-*value of a modified Fisher's exact test for overrepresentation [35]. ^b^Fold enrichment in each gene group compared to the base set. ^c^Gene group with expression that increases with advancing age (same genes used in Table 8, 1430 genes). ^d^Hypergeometric test for underrepresentation using annotated genes from a total set of 13,232 genes as a base population [36].

**Table 8:** Terms in the Gene Ontology and KEGG pathway databases over-represented among genes that increased expression with advancing age in human lymphocytes (1,430 genes in total 13,232).

Category	Term	*P* value^a^	Bonferroni	Fold^b^	Decreasing group^d^	
					Fold^b^	*P* value^e^
GO Bio.Process	signal transduction	2.1(10^−10^)	1.0(10^−6^)	1.34	0.72	8.8(10^−11^)
	immune response	9.2(10^−10^)	4.3(10^−6^)	1.84	1.02	6.0(10^−1^)
	defense response	7.0(10^−9^)	3.3(10^−5^)	1.90	0.76	2.7(10^−2^)
	response to external stimulus	1.3(10^−8^)	6.2(10^−5^)	1.84	0.66	1.4(10^−3^)
	cytoskeleton organization and biogenesis	6.8(10^−7^)	3.2(10^−3^)	1.78	0.58	2.4(10^−4^)
	positive regulation of cellular process	9.6(10^−7^)	4.5(10^−3^)	1.51	0.97	4.1(10^−1^)
	response to wounding	7.2(10^−6^)	3.3(10^−2^)	1.80	0.62	3.0(10^−3^)
	cell adhesion	7.6(10^−6^)	3.5(10^−2^)	1.64	0.71	6.0(10^−3^)
GO Cel.Component	plasma membrane	7.0(10^−11^)	5.5(10^−8^)	1.42	0.62	1.0(10^−13^)
	membrane part	1.1(10^−7^)	8.9(10^−5^)	1.21	0.79	3.5(10^−10^)
	cytoskeleton	1.4(10^−7^)	1.1(10^−4^)	1.56	0.69	1.5(10^−4^)
	integral to plasma membrane	2.3(10^−6^)	1.8(10^−3^)	1.49	0.69	1.0(10^−4^)
GO Mol.Function	protein binding	9.6(10^−10^)	2.4(10^−6^)	1.16	0.93	3.3(10^−3^)
	actin binding	9.1(10^−7^)	2.3(10^−3^)	2.07	0.57	4.4(10^−3^)
	signal transducer activity	1.3(10^−6^)	3.2(10^−3^)	1.37	0.79	4.8(10^−4^)
	GTPase activity	1.4(10^−6^)	3.5(10^−3^)	2.24	0.62	2.7(10^−2^)
	receptor binding	1.5(10^−6^)	3.7(10^−3^)	1.68	0.76	1.8(10^−2^)
	GTP binding	1.5(10^−5^)	3.9(10^−2^)	1.79	0.86	2.0(10^−1^)
KEGG Pathway	Regulation of actin cytoskeleton	3.1(10^−9^)	6.0(10^−7^)	2.44	0.52	6.4(10^−3^)
	Natural killer cell mediated cytotoxicity	1.3(10^−5^)	2.6(10^−3^)	2.25	0.26	2.3(10^−4^)
	Focal adhesion	3.2(10^−5^)	6.4(10^−3^)	2.02	0.55	1.2(10^−2^)

a EASE-score, *P*-value of a modified Fisher's exact test for overrepresentation [35]. ^b^Fold enrichment in each gene group compared to the base set. ^c^Gene group with expression that decreases with advancing age (same genes used in Table 7, 1878 genes). ^e^Hypergeometric test for underrepresentation using annotated genes from a total set of 13,232 genes as a base population [36].

Based upon the identified lists of age-related genes for both brain and lymphocyte samples, we also explored for differences in basic genomic architecture. Our guide for this was a detailed analysis recently presented on characteristics of housekeeping genes [13]. The results of this analysis are presented in Tables 9 and 10, where we document the differences between the up and down-regulated sets in comparison with the characteristics of the non-regulated genes. In summarizing these results, while we found some striking differences, for example with coding sequence length in brain, we note that this was not replicated in lymphocytes. Instead we highlight a single category that does appear to be replicating, namely the ratio of average intron to average exon sequence length. We used second-order factorial ANOVA models to perform combined analyses for all variables. For this, the intron/exon sequence ratio is highly significant (F_2,24158_ = 41.57, P = 9.5×10^−19^) with no evidence of interaction between the two sets (P = 0.69). This can be taken in context with the same analysis for coding sequence length, where the tissue by group (group defined by up, down, or non-regulated genes) interaction term was highly significant (P<0.0001). For all other combined analyses, the main group effect was either non-significant or the interaction term was highly significant (P<0.0001).

**Table 9:** Genomic architecture comparisons for age associated genes in human brain samples.

	Negative asso.^a^	Positive asso.^b^	Unregulated	P-value
	(n = 1,369)	(n = 1,746)	(n = 9,405)	
pre-mRNA length	75652±4560	53683±2018	62344±1186	1.9(10^−1^)
Coding sequence length	1311±28	2057±44	1722±18	7.0(10^−60^)
Number of exons	9.2±0.2	13.0±0.3	11.2±0.1	1.1(10^−22^)
Total intron length	74662±4636	51237±2033	60622±1202	1.4(10^−1^)
5′ UTR length	215±6	227±6	210±2	3.0(10^−2^)
3′ UTR length	1155±34	1219±29	1202±13	3.6(10^−1^)
Average intron of ea. transcript	8651±485	5312±249	6648±144	1.5(10^−9^)
Average exon of ea. transcript	424±14	435±13	429±5	9.9(10^−1^)
Average intron / average exon	28.7±1.5	16.4±0.7	21.9±0.5	5.7(10^−10^)

All data are base pair ±SEM. P-values were calculated from log_10_ transformed data using ANOVA. ^a^Negatively associated genes were those whose expression decreases with age-at-death (P<0.05). ^b^Positively associated genes were those for which expression increased with age-at-death (P<0.05). Unregulated genes were those not significantly correlated with age.

**Table 10:** Genomic architecture comparisons for age associated genes in human lymphocyte samples.

	Negative asso.^a^	Positive asso.^b^	Unregulated	P-value
	(n = 2,364)	(n = 1,980)	(n = 7,898)	
pre-mRNA length	51908±1755	49489±1880	54879±1115	1.2(10^−6^)
Coding sequence length	1724±52	1628±51	1710±18	6.9(10^−3^)
Number of exons	11.5±0.2	10.7±0.2	11.1±0.1	2.1(10^−5^)
Total intron length	49705±1764	47549±1893	53364±1135	7.5(10^−9^)
5′ UTR length	195±4	211±5	208±2	1.0(10^−3^)
3′ UTR length	1104±25	1134±28	1185±15	3.9(10^−4^)
Average intron of ea. transcript	5497±209	5463±256	5651±110	1.5(10^−6^)
Average exon of ea. transcript	381±10	420±11	435±6	7.7(10^−11^)
Average intron / average exon	20.5±1.0	17.7±0.7	19.2±0.4	7.1(10^−10^)

All data are base pair ±SEM. P-values were calculated from log_10_ transformed data using ANOVA. ^a^Negatively associated genes were those whose expression decreases with age (P<0.05). ^b^Positively associated genes were those for which expression increased with age (P<0.05). Unregulated genes were those not significantly correlated with age.

Finally, we used these two samples to pursue the question of whether variance in gene expression itself changes with age. Our hypothesis was that variance might increase with age as a consequence of accumulating somatic mutation [11] and/or increasingly heterogeneous environmental exposures. For the brain sample, an F-test for equality of variances was conducted on all 14,078 transcripts by dividing the sample in two groups according to the median age. We observed only 10 genes that exceeded a strict multiple test correction threshold and in each case there was evidence that the distributions of these genes deviated from normality. There was no overlap in this set for the highest scoring candidates with genes found to change with age in the linear regression analyses. There were 6 cases where an age-related gene also had a borderline significant F-test (P<0.05). This proportion was not significantly different than what could be found in the entire set (not shown). For the lymphocyte set, we took a slightly different approach due to its size and rather than dividing the sample at the median chose to examine decile bins and applied a Levene's robust variance test to explore for differences across age groups. There was again no strong evidence that variance differed across these groupings, the most significant finding being for the *NLP* gene (P = 2.3×10^−6^), and with only 2 genes in total attaining significance after multiple test correction. As a final closing note, we enumerated the number of statistical tests used in this study in its entirety, arriving at an approximate number of 110,000. This may be used as a reference for any of the un-corrected P-values that are presented.

## Discussion

We consider the most important finding in this study to be that expression levels of genes involved in mitochondrial processes are decreasing with age, as originally proposed by Zahn et al. and also supported by Miller et al. [6], [16]. In the former study, a key observation was that this is apparent in multiple tissues as well as in species other than humans. The present analysis provides a confirmation of this as a common characteristic of aging, in that this evident in both brain and lymphocytes. For these two samples there are also tissue specific themes that have emerged that we consider a validation of the quality of the expression phenotypes originally obtained. In the brain sample, consistent with previous results [11], a decrease in genes involved in synaptic function was observed, which follows from the documented changes in synaptic function that occur with age [8]. In lymphocytes, there was evidence that genes involved in the immune response increase expression with age, which might be regarded as a reflection of chronic persisting viruses such as cytomegalovirus (CMV) [17]. While we acknowledge central differences in these samples that include ethnicity, the age ranges, sample size, and the cell types represented, in this discussion we highlight these and other pathways with a comparison of the two different tissues as a guide.

A key distinction in contrasting the study by Zahn et al. was the specific focus on genes of the mitochondrial respiratory chain [6]. While many of these are included in our emergent lists of genes of the mitochondrion (see Tables S1 and S3), results indicate that the effects of age on mitochondrial function may be broader. For example, in the brain the most significant finding across all pathways was for cytoplasm genes (Table 3). Although this categorization includes all genes of the mitochondrion, and indeed our results suggest it is the latter grouping that contributes to the strength of the statistic, there remain additional genes that may be enriched in this set (see Table S1). Oxidative phosphorylation itself did emerge as a highly significant term associated with aging in the brain sample, but not in lymphocytes, indicating that there are likely to be tissue specific differences in the component genes related to mitochondrial function (Tables 3 and 7). Nonetheless, these data together with previous studies provide an intriguing foundation to investigate if this is a primary event in the aging process or derivative of a general decrease in energy metabolism and activity that accompanies old age (e.g. [18]).

That transcript levels are both decreasing and increasing is an important indication that the aging process does not lead to a unidirectional decline in expression. While this was evident in both brain and lymphocytes, in each case the relative fold-enrichment of gene categories was higher among negatively correlated transcripts. Thus, the up-regulated categories consisted of more unique transcripts in general than those that were down-regulated. In brain we noted several pathways that appear to have component genes that increase in expression with advancing age, the foremost among these encompassing genes that encode nuclear proteins. The most significant categorizations that emerged included a large contingent of genes related to DNA-binding and transcription, the most abundant being zinc-finger proteins. Although genes related to transcription were highlighted previously, results suggested a mix of both positive and negative regulation [11]. In contrast however, Miller et al. did observe an overlap of genes related to transcription that were increasing in relation to both ageing and to Alzheimer's disease (AD) [16].

In lymphocytes, immune response was the most significant category associated with increasing expression. However, there are several issues that are impossible to resolve for this latter result, especially regarding which cell types are represented in this sample (e.g. [19]). For example, this might relate to an accumulation of highly differentiated T cells due to acceleration by persistent CMV infection but this would require extensive further study [17]. There were also some interesting highlights among the many individual genes that were up-regulated with age in lymphocytes. In particular the most significant up-regulated gene *IGFBP3* is intriguing in the context that another member of the insulin-like growth factor binding protein family, *IGFBP7* has recently been implicated as an inducer of apoptosis in human melanoma cell lines [20], [21]. *IGFBP3* itself has also been shown to be increased in senescent human fibroblasts [22]. Those findings together with results of the present study provide support for the role of insulin-like growth factor signaling in cellular senescence.

For the brain sample, we were intrigued by the emergent pattern that expression of cytoplasm genes may be decreasing with age, while expression of nuclear genes may be increasing with age. While this might reflect the large number of mitochondrial and transcription genes in these particular sets, we cannot ignore the possibility that a basic change in cell morphology might be at play. One explanation may be that neuronal number remains stable with age, but synaptic vesicle density decreases [7], [8]. This relative change might give rise to our observations here. In support of this, genes related to neuronal projections (which includes dendrite genes) and synapse genes are major categories that appear to decrease with age (see Table 3). Also in support of this, the highest ranking individual gene, *SVOP*, encoding the SV2 related protein, is localized to the synaptic vesicle and appears to be an ion transporter. We had anticipated that more neuronal specific genes would exhibit a decreasing pattern, but noted that even some common neuronal reference genes, such as *ENO2* (ranking 7597^th^), were not associated with age. Thus, in contrast to the enrichment of neuronal projection genes mentioned above (and see Table 3), this might support the concept that a decrease in neuronal number is not a major feature of aging [23]. A potentially important source of confounding in analyses of post-mortem brain samples is the mode of death, which we have not examined in more detail in this study. In particular, agonal state and pH have been highlighted as contributors to brain mRNA expression [24]–[26]. The present study has strong similarities with the study by Li et al., (2004)[25]. However, the relationship between pH and age was previously explored, but there was no evidence of a significant correlation [24], [27]. Interestingly, although it was a small sample Vawter et al. also noted significant correlations of age with mitochondrial genes regardless of agonal state (see specifically supplementary table 4 from [24]). Nonetheless, one explanation might be that transcription factor activation and mitochondrial deactivation is a natural response to hypoxic stress which may be more common in elderly individuals (e.g. [25], [28]). Another possibility might be that both aging and extended hypoxia dependent on the mode of death share similarities in terms of gene regulation.

For the lymphocyte sample, the most significantly enriched terms were found in the class of negatively regulated genes, where the ribosomal and translation machinery appears to be strongly affected by age. This could potentially contribute to “immunosenescence”, whereby the immune system decays with time, rendering elderly individuals more susceptible to infectious diseases [29]. While changes at the expression level for this class of genes have to our knowledge not been previously reported on this scale, the concept that protein synthesis is involved in immunosenescence is not new [30]. This result is in our view the most striking difference between lymphocytes and brain. Although this suggests a more general decreases in metabolism in aging lymphocytes, we consider this an important reminder that tissue specificity can play a major role in gene expression profiling [31].

Another important feature of this study is the relative degree of stability in gene expression across the age spectrum. To put this in context, the strongest effects of age on any genes were 18% variance explained for *TAC3* in brain and 29% for *LRRN3* in lymphocytes, but in examining Tables 1, 2, 5 and 6, it is apparent that effect sizes drop off rapidly. For each of these top-ranked genes there was an approximately 2-fold change in average expression levels over the age ranges. This is comparable to well documented changes in hormone levels with age, such as for IGF1 [2], [32]. Also of note, genes claimed to be changing with age previously exhibited differences between young and older groups in the range of 2–3 fold [11]. This again might suggest that cell-loss and/or changes in organ function/morphology are more important than broad changes in gene expression in the decrease in health that typically accompanies increasing age. The possibility however cannot be excluded that relatively small changes in gene expression have a large impact on cell function. As an example, disorders such as Parkinson's disease (PD) can be caused by simple gene dosage effects, as has now been shown for the *SNCA* gene which encodes alpha-synuclein [33]. Although rare, we noted several genes that may be changing to a larger degree, but which fell below a strict significance threshold, for example *ZIC3* in brain. However, there were no similar cases in the much more powerful lymphocyte sample.

Finally, we also explored basic gene architecture characteristics in an attempt to provide some insight into why expression might vary with age. The only parameter that emerged as significant and equivalent in both brain and lymphocytes was the intron/exon length ratio, this representing a metric of gene “compactness” [13]. This might be interpreted as suggesting that non-compact genes are more susceptible to mutations that disrupt regulation, and thus lead to decreasing expression with advancing age. However, we cannot exclude the possibility that what we are seeing is simply a result of the gene pathways that are over-represented, these being enriched for compact or non-compact genes. In other words, if specific pathways are affected by age, the genes that represent those pathways may have similar features. We consider this nonetheless an intriguing finding in that it is replicating in different tissues.

In summary, we validate previous findings that a decrease in mitochondrial gene expression appears to be a common theme in the aging process. Whether this is a primary event that causes a decline in health with advancing age or a result of a general decrease in metabolism in the elderly remains a topic for further investigation. We also highlight additional novel pathways that may be age dependent but with dramatic differences between tissues, in particular with genes related to transcription and to translation. These results may provide a valuable foundation for understanding the molecular consequences of aging and emphasize the development of catalogues of senescence-related genes in additional tissues.

## Materials and Methods

### Human Samples

The primary sample used in this study consisted of 191 individuals with ages-at-death data ranging from 65–100 years for which brain autopsy tissue was obtained. Expression level data were obtained using the Illumina HumanRefSeq-8 expression BeadChip platform for a total of 14,078 transcripts in which expression was detected in 5 or more individuals. Detailed descriptions of the human samples as well as the expression profiling protocol are provided in the original publication for which this sample was presented [12]. To create a working dataset, expression phenotypes and covariate data were merged that included age-at-death, post-mortem interval (pmi), gender, brain region, and transcript detection rates. From this, we generated 2 additional covariates for 1) total average expression level from all transcripts and 2) total average expression level for all transcripts that were detected in all individuals (5269 transcripts fulfilled the latter criteria). All individual transcript levels, the global average transcript levels, and pmi were log_10_ transformed prior to inclusion in analyses.

The second sample consisted of 1240 individuals ranging in age from 15-94 in which fresh blood lymphocytes had been obtained and mRNA extracted. Expression phenotyping was conducted using the Illumina Sentrix Human Whole Genome (WG-6) Series I BeadChip platform. In total there were 19,648 individual transcript measures, representing approximately 18,500 genes. Details of the sample and expression protocols have been provided previously in the original publication describing this sample [15]. For our analysis, we used the normalized expression phenotypes without further transformation.

### Correlation of Age and Transcript Level

We classified the extremes as outliers that are expected to be observed once or more in 1930 individual log transformed transcript estimates with assumption of independence and normal distribution of 193 measures for each transcript.
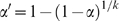
where *α′* is the alpha level for each measure, *α* is 0.05 and *k* 193. Based on normal distribution assumption,
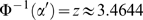
where Φ is standard normal cumulative distribution fuction, z is z-score of each measure.

The normality of log transformed observed values for each transcript was tested by means of a Shapiro-Wilk W test. To assess differences of transcript detection rates or global expression levels across brain regions, as well as to test differences in characteristics of genomic architecture between age-related gene sets, ANOVA was used. Contingency tables (for example comparing the proportions of housekeeping genes) were evaluated by means of a standard chi-square test. All statistical analyses not related to pathway based tests were performed using STATA se 9.0. For the brain sample, the dependency of expression level on age-at-death was tested by fitting a linear regression model for each transcript:
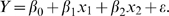
where *Y* is the log_10_ transformed expression level of the transcript, *x*_1_ age-at-death, *x*_2_ log_10_ of global expression, *β_0_* intercept, *β_1_* and *β_2_* slope of each independent variable, and *ε* random error. For the lymphocyte sample, the same test was applied but without the global expression covariate according to the following:
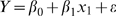
where *Y* is the normalized expression level of each transcript, *x*_1_ age, *β_0_* intercept, *β_1_* slope of age, and *ε* random error. We also allowed for non-independence of expression measures given the relatedness of family members in the lymphocyte sample [34].

### Gene Ontology and KEGG Pathway Analyses

The Entrez Gene IDs, symbols, and descriptions of genes for all tested transcripts were attained by Entrez Programming Utilities (eUtils) using GI number with the aid of a Perl script. This facilitated a search for replaced sequence identifiers and extracted information of interest by scanning the output text files from Entrez eUtils on the transcripts. Using the attained Entrez Gene IDs as identifiers of genes, we obtained the total lists of both Gene Ontology terms and KEGG pathway descriptors, with which our selected set of genes was annotated and analyzed using the DAVID functional annotation tool [14] with the most recent data update (January, 2008). The EASE scores [35] from the DAVID tool were used in trimming to over-represented term lists, with Bonferroni corrections of the scores from the tool as an inclusion criteria of Tables 3, 4, 7 and 8. We scrutinized ancestor-descendant relationships in the Gene Ontology structure among the terms in the enrichment lists on the basis of *is_a*, *part_of*, and *regulates* relationship by scanning the master ontology file which was updated in April, 2008. Our goal with this was to determine if ancestor terms had emerged as significant primarily because of enrichment of one of their descendant terms. We detail our strategy for this in Figure S1. Among the GO terms in the list, those which had no descendants were labeled as ‘end-terms’. For every term excluding end-terms, we created the artificial descendants. These were intended to represent the complement of the set of genes annotated with a descendant term in the list with respect to all genes in the ancestor. The artificial term for every descendant of every ancestor in the list was then tested to determine if it was over-represented by applying a hypergeometric test [36]. The ancestors for which all artificial descendant terms were over-represented at P<0.005 were labeled ‘significant ancestor terms’. The terms whose artificial descendants for end-terms or significant ancestors were over-represented were added to the set of ‘significant ancestor terms’. After modifying the set, the terms in the set were checked if they fulfilled inclusion criteria (enriched complement at P<0.005) and the set was updated iteratively until there was no change. The end-terms and significant ancestor terms are listed in Tables S1, S2, S3, S4. The remaining terms in the enrichment list follow in the same tables that are significantly over-represented mainly by a highly enriched descendant. P-values of the enrichment test for the artificial terms are also shown in the Tables S1, S2, S3, S4.

### Genomic Architecture of Age-related Gene Sets

The genomic positions of start and end points of transcripts, their coding sequences, and their exons and introns in both brain and lymphocyte gene sets were collected from the UCSC genome browser using Genbank accession numbers (e.g. NM_018711) as identifiers. Information on some of the transcripts that were detected in both samples was not available from the UCSC browser due to the records being suppressed. Therefore, the number of transcripts in the genomic architecture analysis was reduced to 12,520 and 12,242 for brain and lymphocyte samples, respectively. Based on the positional data, lengths of pre-mRNA sequences, coding sequence, total intron, 5′UTR, 3′UTR, average intron and average exon of each transcript, as well as the number of exons and the ratio of intron per coding sequence length were calculated and transformed on a log_10_ scale. For comparative analyses of gene characteristics, the transcripts were divided into 3 groups, up-regulated genes (positively correlated with age at a P<0.05 threshold), down-regulated (negatively correlated with age at a P<0.05 threshold), and a set of genes that were not significantly altered over the age spectrum. Differences between groups were assessed using ANOVA. Combined analyses were performed on both sets using second order factorial ANOVA with sample source as a covariate.

### URLs

Entrez Gene: http://www.ncbi.nlm.nih.gov/sites/entrezdbgene; Entrez eUtils: http://eutils.ncbi.nlm.nih.gov/entrez/eutils; DAVID 2008: http://david.abcc.ncifcrf.gov/; Gene Ontology (GO): http://www.geneontology.org/; UCSC genome browser: http://genome.ucsc.edu/

## Supporting Information

Figure S1 Overrepresented GO term analysis
(0.06 MB PDF)

Table S1 Terms in the Gene Ontology and KEGG pathway databases enriched among genes that decreased expression with advancing age in brain (1450 genes in total 13,216)
(0.15 MB PDF)

Table S2 Terms in the Gene Ontology and KEGG pathway databases enriched among genes that increased expression with advancing age in brain (1943 genes in total 13,216)
(0.09 MB PDF)

Table S3 Terms in the Gene Ontology and KEGG pathway databases enriched among genes that decreased expression with advancing age in the human lymphocytes (1878 genes in total 13,232)
(0.13 MB PDF)

Table S4 Terms in the Gene Ontology and KEGG pathway databases enriched among genes that increased expression with advancing age in the human lymphocytes (1430 genes in total 13,232)
(0.12 MB PDF)
