# Supplementary material for: Transcriptome-Wide Assessment of Human Brain and Lymphocyte Senescence
Source: PLoS One. 2008 Aug 20;3(8):e3024. doi: 10.1371/journal.pone.0003024 (PMC2515343; doi:10.1371/journal.pone.0003024)
Supplement: Figure S1 — Overrepresented GO term analysis (0.06 MB PDF) [file pone.0003024.s001.pdf]

Figure S1. Over-represented GO term analysis

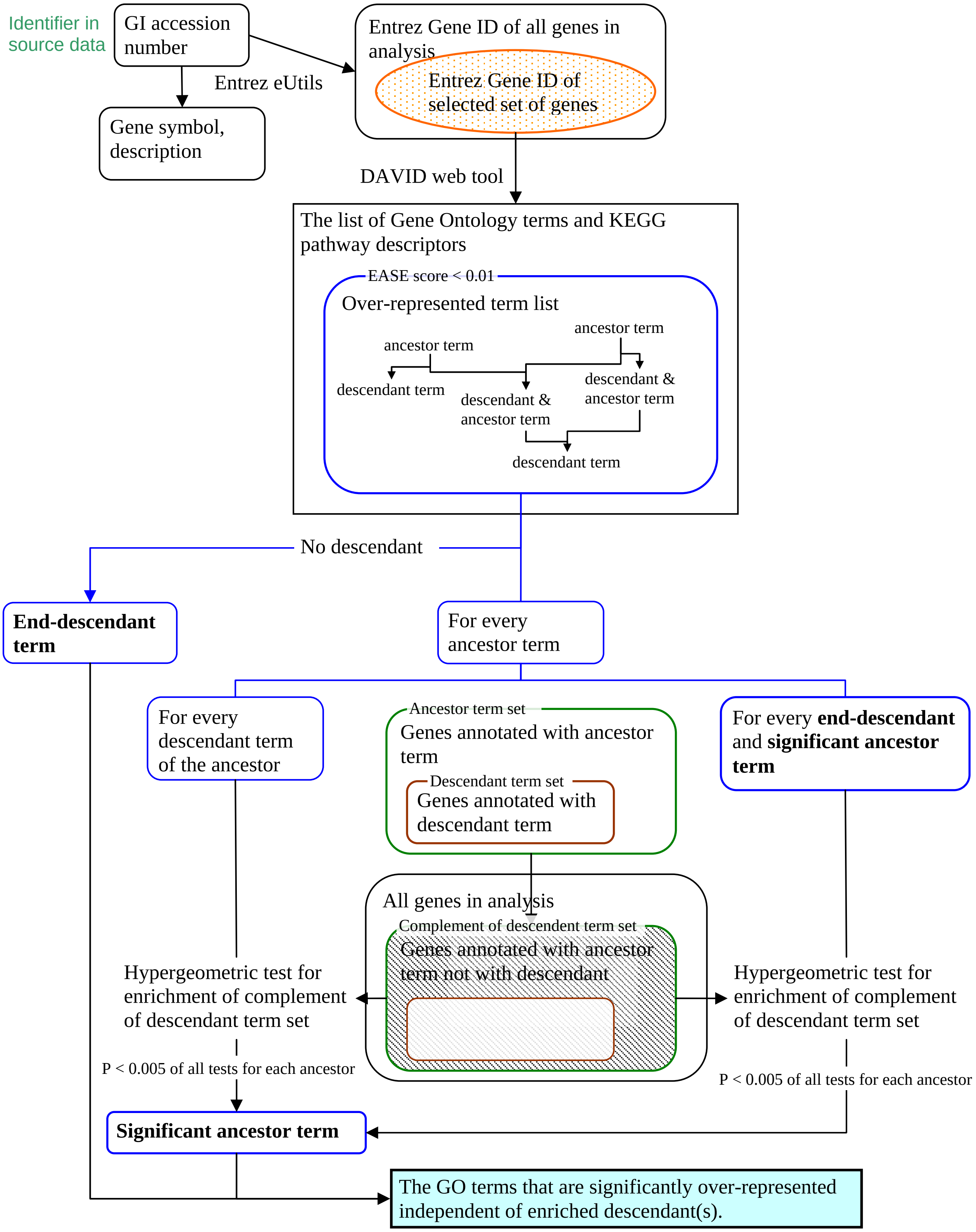

Blue rounded rectangles indicate that only the terms in the Over-represented term list are considered at the step.
