# Supplementary material for: Transcriptome-Wide Assessment of Human Brain and Lymphocyte Senescence
Source: PLoS One. 2008 Aug 20;3(8):e3024. doi: 10.1371/journal.pone.0003024 (PMC2515343; doi:10.1371/journal.pone.0003024)
Supplement: Table S1 — Terms in the Gene Ontology and KEGG pathway databases enriched among genes that decreased expression with advancing age in brain (1450 genes in total 13,216) (0.15 MB PDF) [file pone.0003024.s002.pdf]

Table S1. Terms in the Gene Ontology and KEGG pathway databases enriched among genes that decreased expression with advancing age in brain (1450 genes in total 13,216)

Category	Term	Count	%Hit[a]	PValue[b]	Bonferroni	HGeom[c]	Fold[d]	Fold-U[e]	HGe-U[f]
GO_BP	nervous system development	108	19%	$7.8(10^{-09})$	$3.8(10^{-05})$	$4.1(10^{-09})$	1.73	0.76	$6.7(10^{-03})$
	except neurite development	78				$3.0(10^{-05})$	1.56		
GO_BP	synaptic transmission	50	23%	$7.4(10^{-07})$	$3.5(10^{-03})$	$3.1(10^{-07})$	2.07	0.28	$3.1(10^{-07})$
GO_BP	oxidative phosphorylation	28	31%	$1.4(10^{-06})$	$6.7(10^{-03})$	$4.0(10^{-07})$	2.73	0.15	$5.3(10^{-05})$
	except ATP synthesis coupled proton transport	15				$4.4(10^{-04})$	2.56		
	except mitochondrial electron transport, NADH to ubiquinone	15				$2.7(10^{-04})$	2.66		
GO_BP	neurite development	30	27%	$1.4(10^{-05})$	$6.5(10^{-02})$	$4.9(10^{-06})$	2.35	0.41	$3.2(10^{-03})$
	except axonogenesis	7				$3.4(10^{-03})$	3.27		
	except dendrite development	24				$2.8(10^{-04})$	2.11		
GO_BP	coenzyme metabolic process	34	23%	$8.2(10^{-05})$	$3.3(10^{-01})$	$3.5(10^{-05})$	2.04	0.32	$6.5(10^{-05})$
	except tricarboxylic acid cycle	26				$1.6(10^{-03})$	1.83		
	except acyl-CoA metabolic process	29				$8.0(10^{-04})$	1.84		
GO_BP	G-protein coupled receptor protein signaling pathway	63	18%	$1.0(10^{-04})$	$3.9(10^{-01})$	$5.8(10^{-05})$	1.62	0.76	$2.9(10^{-02})$
	except G-protein signaling, coupled to cAMP nucleotide second messenger	49				$3.1(10^{-03})$	1.48		
GO_BP	transport	250	14%	$4.1(10^{-04})$	$8.6(10^{-01})$	$3.3(10^{-04})$	1.21	0.87	$3.9(10^{-03})$
	except microtubule-based movement	233				$3.3(10^{-03})$	1.17		
	except ATP synthesis coupled proton transport	237				$3.0(10^{-03})$	1.17		
	except zinc ion transport	243				$1.3(10^{-03})$	1.18		
GO_BP	axonogenesis	23	24%	$6.1(10^{-04})$	$9.5(10^{-01})$	$2.3(10^{-04})$	2.17	0.43	$8.7(10^{-03})$
GO_BP	ATP synthesis coupled proton transport	13	33%	$8.5(10^{-04})$	$9.8(10^{-01})$	$2.2(10^{-04})$	2.96	0.34	$5.5(10^{-02})$
GO_BP	mitochondrial electron transport, NADH to ubiquinone	13	32%	$1.4(10^{-03})$	$1.0(10^{-00})$	$3.8(10^{-04})$	2.81	0.00	$1.3(10^{-03})$
GO_BP	neuron recognition	6	67%	$1.5(10^{-03})$	$1.0(10^{-00})$	$1.3(10^{-04})$	5.91	0.00	$2.3(10^{-01})$
GO_BP	glycolysis	12	32%	$2.4(10^{-03})$	$1.0(10^{-00})$	$6.6(10^{-04})$	2.80	0.53	$1.6(10^{-01})$
GO_BP	G-protein signaling, coupled to cAMP nucleotide second messenger	14	28%	$2.9(10^{-03})$	$1.0(10^{-00})$	$9.3(10^{-04})$	2.48	0.40	$4.6(10^{-02})$
GO_BP	microtubule-based movement	17	24%	$4.1(10^{-03})$	$1.0(10^{-00})$	$1.6(10^{-03})$	2.15	0.76	$2.6(10^{-01})$
GO_BP	neuron adhesion	5	71%	$4.2(10^{-03})$	$1.0(10^{-00})$	$3.1(10^{-04})$	6.33	0.00	$3.2(10^{-01})$
GO_BP	dicarboxylic acid metabolic process	6	55%	$4.6(10^{-03})$	$1.0(10^{-00})$	$5.7(10^{-04})$	4.84	0.61	$4.9(10^{-01})$
GO_BP	zinc ion transport	7	44%	$6.0(10^{-03})$	$1.0(10^{-00})$	$1.0(10^{-03})$	3.88	0.83	$5.6(10^{-01})$
GO_BP	dendrite development	6	50%	$7.2(10^{-03})$	$1.0(10^{-00})$	$1.0(10^{-03})$	4.43	0.56	$4.4(10^{-01})$
GO_BP	acyl-CoA metabolic process	5	63%	$7.7(10^{-03})$	$1.0(10^{-00})$	$7.6(10^{-04})$	5.54	0.83	$6.6(10^{-01})$
GO_BP	tricarboxylic acid cycle	8	36%	$8.4(10^{-03})$	$1.0(10^{-00})$	$1.9(10^{-03})$	3.22	0.30	$1.4(10^{-01})$
GO_BP	generation of precursor metabolites and energy	78	19%	$2.4(10^{-06})$	$1.1(10^{-02})$	$1.3(10^{-06})$	1.70	0.74	$1.2(10^{-02})$
	except oxidative phosphorylation	50				$8.0(10^{-03})$	1.40		
	except tricarboxylic acid cycle	70				$2.9(10^{-05})$	1.61		
GO_BP	neuron development	33	25%	$1.5(10^{-05})$	$7.1(10^{-02})$	$5.8(10^{-06})$	2.23	0.51	$8.2(10^{-03})$
	except neurite development	3				$3.3(10^{-01})$	1.48		
GO_BP	transmission of nerve impulse	51	21%	$2.1(10^{-05})$	$9.5(10^{-02})$	$9.9(10^{-06})$	1.84	0.38	$3.6(10^{-06})$
	except synaptic transmission	1				$9.8(10^{-01})$	0.28		
GO_BP	localization	295	14%	$3.9(10^{-05})$	$1.7(10^{-01})$	$3.0(10^{-05})$	1.22	0.87	$1.6(10^{-03})$
	except transport	45				$4.0(10^{-02})$	1.30		
GO_BP	neuron differentiation	38	22%	$5.4(10^{-05})$	$2.3(10^{-01})$	$2.4(10^{-05})$	1.98	0.63	$2.1(10^{-02})$
	except neurite development	8				$3.1(10^{-01})$	1.24		
GO_BP	proton transport	18	30%	$2.3(10^{-04})$	$6.7(10^{-01})$	$6.8(10^{-05})$	2.66	0.67	$1.9(10^{-01})$
	except ATP synthesis coupled proton transport	5				$8.0(10^{-02})$	2.11		
GO_BP	hydrogen transport	18	30%	$2.3(10^{-04})$	$6.7(10^{-01})$	$6.8(10^{-05})$	2.66	0.67	$1.9(10^{-01})$
	except ATP synthesis coupled proton transport	5				$8.0(10^{-02})$	2.11		
GO_BP	establishment of localization	258	14%	$2.4(10^{-04})$	$6.8(10^{-01})$	$1.9(10^{-04})$	1.21	0.87	$3.7(10^{-03})$
	except transport	8				$2.1(10^{-01})$	1.39		
GO_BP	generation of neurons	40	20%	$2.9(10^{-04})$	$7.5(10^{-01})$	$1.4(10^{-04})$	1.80	0.68	$3.0(10^{-02})$
	except neurite development	10				$4.8(10^{-01})$	1.06		
GO_BP	purine ribonucleoside triphosphate biosynthetic process	17	30%	$3.2(10^{-04})$	$7.8(10^{-01})$	$9.2(10^{-05})$	2.69	0.48	$6.3(10^{-02})$
	except ATP synthesis coupled proton transport	4				$1.2(10^{-01})$	2.09		
GO_BP	purine nucleoside triphosphate biosynthetic process	17	30%	$3.2(10^{-04})$	$7.8(10^{-01})$	$9.2(10^{-05})$	2.69	0.48	$6.3(10^{-02})$
	except ATP synthesis coupled proton transport	4				$1.2(10^{-01})$	2.09		
GO_BP	neurite morphogenesis	25	25%	$3.2(10^{-04})$	$7.8(10^{-01})$	$1.2(10^{-04})$	2.17	0.46	$9.9(10^{-03})$
	except axonogenesis	2				$2.3(10^{-01})$	2.22		
GO_BP	neuron morphogenesis during differentiation	25	25%	$3.2(10^{-04})$	$7.8(10^{-01})$	$1.2(10^{-04})$	2.17	0.46	$9.9(10^{-03})$
	except axonogenesis	2				$2.3(10^{-01})$	2.22		

GO_BP	cell-cell signaling	69	17%	$3.4(10^{-04})$	$8.0(10^{-01})$	$2.0(10^{-04})$	1.52	0.40	$8.2(10^{-09})$
	except synaptic transmission	19				$7.3(10^{-01})$	0.90		
GO_BP	ribonucleoside triphosphate biosynthetic process	17	30%	$3.9(10^{-04})$	$8.5(10^{-01})$	$1.2(10^{-04})$	2.64	0.47	$5.7(10^{-02})$
	except ATP synthesis coupled proton transport	4				$1.4(10^{-01})$	1.97		
GO_BP	cofactor metabolic process	37	21%	$4.0(10^{-04})$	$8.5(10^{-01})$	$1.9(10^{-04})$	1.82	0.37	$5.9(10^{-05})$
	except coenzyme metabolic process	3				$7.2(10^{-01})$	0.83		
GO_BP	neurological system process	77	16%	$6.0(10^{-04})$	$9.4(10^{-01})$	$3.8(10^{-04})$	1.46	0.68	$1.3(10^{-03})$
	except synaptic transmission	27				$6.7(10^{-01})$	0.94		
GO_BP	nucleoside triphosphate biosynthetic process	17	29%	$6.0(10^{-04})$	$9.4(10^{-01})$	$1.9(10^{-04})$	2.55	0.57	$1.1(10^{-01})$
	except ATP synthesis coupled proton transport	4				$1.8(10^{-01})$	1.77		
GO_BP	ATP biosynthetic process	14	33%	$6.3(10^{-04})$	$9.5(10^{-01})$	$1.7(10^{-04})$	2.89	0.31	$3.4(10^{-02})$
	except ATP synthesis coupled proton transport	1				$3.8(10^{-01})$	2.22		
GO_BP	nucleoside phosphate metabolic process	14	33%	$6.3(10^{-04})$	$9.5(10^{-01})$	$1.7(10^{-04})$	2.89	0.31	$3.4(10^{-02})$
	except ATP synthesis coupled proton transport	1				$3.8(10^{-01})$	2.22		
GO_BP	cell projection morphogenesis	34	20%	$7.7(10^{-04})$	$9.7(10^{-01})$	$3.7(10^{-04})$	1.82	0.76	$1.2(10^{-01})$
	except neurite development	4				$8.6(10^{-01})$	0.67		
GO_BP	cell part morphogenesis	34	20%	$7.7(10^{-04})$	$9.7(10^{-01})$	$3.7(10^{-04})$	1.82	0.76	$1.2(10^{-01})$
	except neurite development	4				$8.6(10^{-01})$	0.67		
GO_BP	cell projection organization and biogenesis	34	20%	$7.7(10^{-04})$	$9.7(10^{-01})$	$3.7(10^{-04})$	1.82	0.76	$1.2(10^{-01})$
	except neurite development	4				$8.6(10^{-01})$	0.67		
GO_BP	neurogenesis	41	19%	$8.2(10^{-04})$	$9.8(10^{-01})$	$4.3(10^{-04})$	1.70	0.69	$2.8(10^{-02})$
	except neurite development	11				$6.0(10^{-01})$	0.97		
GO_BP	cellular morphogenesis during differentiation	25	23%	$8.9(10^{-04})$	$9.9(10^{-01})$	$3.7(10^{-04})$	2.03	0.49	$1.2(10^{-02})$
	except axonogenesis	2				$5.2(10^{-01})$	1.18		
GO_BP	cellular localization	109	15%	$9.9(10^{-04})$	$9.9(10^{-01})$	$7.2(10^{-04})$	1.34	0.85	$4.1(10^{-02})$
	except microtubule-based movement	92				$1.3(10^{-02})$	1.25		
GO_BP	establishment of cellular localization	107	15%	$1.0(10^{-03})$	$9.9(10^{-01})$	$7.4(10^{-04})$	1.34	0.86	$5.4(10^{-02})$
	except microtubule-based movement	90				$1.3(10^{-02})$	1.25		
GO_BP	ribonucleotide biosynthetic process	19	25%	$1.4(10^{-03})$	$1.0(10^{-00})$	$5.0(10^{-04})$	2.25	0.53	$5.4(10^{-02})$
	except ATP synthesis coupled proton transport	6				$2.1(10^{-01})$	1.48		
GO_BP	purine ribonucleoside triphosphate metabolic process	17	27%	$1.6(10^{-03})$	$1.0(10^{-00})$	$5.4(10^{-04})$	2.35	0.52	$6.8(10^{-02})$
	except ATP synthesis coupled proton transport	4				$3.1(10^{-01})$	1.42		
GO_BP	purine ribonucleotide biosynthetic process	18	26%	$1.6(10^{-03})$	$1.0(10^{-00})$	$5.7(10^{-04})$	2.28	0.57	$8.4(10^{-02})$
	except ATP synthesis coupled proton transport	5				$2.7(10^{-01})$	1.43		
GO_BP	nucleoside triphosphate metabolic process	18	26%	$1.6(10^{-03})$	$1.0(10^{-00})$	$5.7(10^{-04})$	2.28	0.57	$8.4(10^{-02})$
	except ATP synthesis coupled proton transport	5				$2.7(10^{-01})$	1.43		
GO_BP	purine nucleotide biosynthetic process	19	25%	$1.6(10^{-03})$	$1.0(10^{-00})$	$6.0(10^{-04})$	2.22	0.53	$5.0(10^{-02})$
	except ATP synthesis coupled proton transport	6				$2.3(10^{-01})$	1.44		
GO_BP	ribonucleoside triphosphate metabolic process	17	26%	$1.9(10^{-03})$	$1.0(10^{-00})$	$6.6(10^{-04})$	2.32	0.51	$6.2(10^{-02})$
	except ATP synthesis coupled proton transport	4				$3.4(10^{-01})$	1.36		
GO_BP	purine nucleoside triphosphate metabolic process	17	26%	$1.9(10^{-03})$	$1.0(10^{-00})$	$6.6(10^{-04})$	2.32	0.51	$6.2(10^{-02})$
	except ATP synthesis coupled proton transport	4				$3.4(10^{-01})$	1.36		
GO_BP	biosynthetic process	149	14%	$2.0(10^{-03})$	$1.0(10^{-00})$	$1.5(10^{-03})$	1.25	0.83	$8.4(10^{-03})$
	except ATP synthesis coupled proton transport	136				$1.6(10^{-02})$	1.19		
GO_BP	organelle ATP synthesis coupled electron transport	14	29%	$2.4(10^{-03})$	$1.0(10^{-00})$	$7.5(10^{-04})$	2.53	0.00	$3.5(10^{-04})$
	except mitochondrial electron transport, NADH to ubiquinone	1				$6.2(10^{-01})$	1.11		
GO_BP	cytoskeleton-dependent intracellular transport	20	23%	$2.8(10^{-03})$	$1.0(10^{-00})$	$1.2(10^{-03})$	2.06	0.78	$2.4(10^{-01})$
	except microtubule-based movement	3				$2.7(10^{-01})$	1.66		
GO_BP	ATP synthesis coupled electron transport	14	28%	$2.9(10^{-03})$	$1.0(10^{-00})$	$9.3(10^{-04})$	2.48	0.00	$2.9(10^{-04})$
	except mitochondrial electron transport, NADH to ubiquinone	1				$6.6(10^{-01})$	0.99		
GO_BP	cellular respiration	12	31%	$3.0(10^{-03})$	$1.0(10^{-00})$	$8.5(10^{-04})$	2.73	0.34	$5.5(10^{-02})$
	except tricarboxylic acid cycle	4				$1.2(10^{-01})$	2.09		
GO_BP	cAMP-mediated signaling	15	27%	$3.0(10^{-03})$	$1.0(10^{-00})$	$1.0(10^{-03})$	2.37	0.48	$6.3(10^{-02})$
	except G-protein signaling, coupled to cAMP nucleotide second messenger	1				$5.1(10^{-01})$	1.48		
GO_BP	nucleobase, nucleoside and nucleotide metabolic process	35	19%	$3.1(10^{-03})$	$1.0(10^{-00})$	$1.7(10^{-03})$	1.66	0.68	$3.4(10^{-02})$
	except ATP synthesis coupled proton transport	22				$1.1(10^{-01})$	1.32		
GO_BP	ATP metabolic process	14	27%	$3.5(10^{-03})$	$1.0(10^{-00})$	$1.2(10^{-03})$	2.43	0.39	$4.1(10^{-02})$
	except ATP synthesis coupled proton transport	1				$7.6(10^{-01})$	0.74		
GO_BP	system process	92	15%	$3.6(10^{-03})$	$1.0(10^{-00})$	$2.6(10^{-03})$	1.32	0.82	$2.9(10^{-02})$
	except synaptic transmission	42				$7.3(10^{-01})$	0.92		
GO_BP	cellular carbohydrate metabolic process	42	18%	$3.8(10^{-03})$	$1.0(10^{-00})$	$2.2(10^{-03})$	1.56	0.87	$2.2(10^{-01})$
	except glycolysis	30				$6.6(10^{-02})$	1.32		
GO_BP	nucleotide metabolic process	33	19%	$4.1(10^{-03})$	$1.0(10^{-00})$	$2.2(10^{-03})$	1.66	0.68	$4.2(10^{-02})$
	except ATP synthesis coupled proton transport	20				$1.4(10^{-01})$	1.29		
GO_BP	cell communication	301	13%	$4.2(10^{-03})$	$1.0(10^{-00})$	$3.6(10^{-03})$	1.14	0.97	$2.4(10^{-01})$
	except synaptic transmission	251				$2.2(10^{-01})$	1.04		
	except G-protein coupled receptor protein signaling pathway	238				$1.7(10^{-01})$	1.05		
GO_BP	purine ribonucleotide metabolic process	18	23%	$5.3(10^{-03})$	$1.0(10^{-00})$	$2.2(10^{-03})$	2.05	0.60	$8.6(10^{-02})$
	except ATP synthesis coupled proton transport	5				$4.5(10^{-01})$	1.14		
GO_BP	ribonucleotide metabolic process	19	22%	$5.8(10^{-03})$	$1.0(10^{-00})$	$2.5(10^{-03})$	1.98	0.63	$9.3(10^{-02})$
	except ATP synthesis coupled proton transport	6				$4.2(10^{-01})$	1.16		
GO_BP	purine nucleotide metabolic process	19	22%	$5.8(10^{-03})$	$1.0(10^{-00})$	$2.5(10^{-03})$	1.98	0.55	$4.8(10^{-02})$
	except ATP synthesis coupled proton transport	6				$4.2(10^{-01})$	1.16		

GO_BP	cellular biosynthetic process	112	14%	$6.1(10^{-03})$	$1.0(10^{-00})$	$4.5(10^{-03})$	1.26	0.81	$1.1(10^{-02})$
	except ATP synthesis coupled proton transport	99				$4.6(10^{-02})$	1.17		
GO_BP	cofactor catabolic process	9	35%	$6.2(10^{-03})$	$1.0(10^{-00})$	$1.5(10^{-03})$	3.07	0.26	$8.2(10^{-02})$
	except tricarboxylic acid cycle	1				$3.8(10^{-01})$	2.22		
GO_BP	aerobic respiration	10	31%	$7.2(10^{-03})$	$1.0(10^{-00})$	$2.0(10^{-03})$	2.77	0.21	$3.7(10^{-02})$
	except tricarboxylic acid cycle	2				$3.1(10^{-01})$	1.77		
GO_BP	nucleotide biosynthetic process	24	20%	$7.2(10^{-03})$	$1.0(10^{-00})$	$3.6(10^{-03})$	1.77	0.61	$4.2(10^{-02})$
	except ATP synthesis coupled proton transport	11				$3.0(10^{-01})$	1.20		
GO_BP	cyclic-nucleotide-mediated signaling	17	23%	$7.3(10^{-03})$	$1.0(10^{-00})$	$3.0(10^{-03})$	2.04	0.63	$1.2(10^{-01})$
	except G-protein signaling, coupled to cAMP nucleotide second messenger	3				$5.2(10^{-01})$	1.11		
GO_BP	cytoskeleton organization and biogenesis	61	16%	$7.4(10^{-03})$	$1.0(10^{-00})$	$5.0(10^{-03})$	1.38	1.08	$7.7(10^{-01})$
	except microtubule-based movement	44				$9.7(10^{-02})$	1.22		
GO_BP	phosphorylation	87	15%	$7.8(10^{-03})$	$1.0(10^{-00})$	$5.8(10^{-03})$	1.30	0.93	$2.6(10^{-01})$
	except oxidative phosphorylation	59				$4.0(10^{-01})$	1.04		
GO_BP	acetyl-CoA metabolic process	9	33%	$7.9(10^{-03})$	$1.0(10^{-00})$	$2.0(10^{-03})$	2.96	0.49	$2.1(10^{-01})$
	except tricarboxylic acid cycle	1				$4.5(10^{-01})$	1.77		
GO_BP	monovalent inorganic cation transport	38	17%	$8.0(10^{-03})$	$1.0(10^{-00})$	$4.7(10^{-03})$	1.53	0.85	$2.0(10^{-01})$
	except ATP synthesis coupled proton transport	25				$1.7(10^{-01})$	1.22		
GO_BP	microtubule-based process	28	19%	$8.3(10^{-03})$	$1.0(10^{-00})$	$4.4(10^{-03})$	1.67	0.90	$3.5(10^{-01})$
	except microtubule-based movement	11				$2.7(10^{-01})$	1.23		
GO_BP	acetyl-CoA catabolic process	8	36%	$8.4(10^{-03})$	$1.0(10^{-00})$	$1.9(10^{-03})$	3.22	0.30	$1.4(10^{-01})$
	except tricarboxylic acid cycle	0				$1.0(10^{-00})$	1.00		
GO_CC	cytoplasmic part	421	15%	$9.0(10^{-11})$	$7.2(10^{-08})$	$6.8(10^{-11})$	1.29	0.76	$4.2(10^{-11})$
	except mitochondrion	270				$4.2(10^{-03})$	1.15		
	except synaptic vesicle	406				$3.4(10^{-09})$	1.26		
	except proteasome core complex (sensu Eukaryota)	413				$7.8(10^{-10})$	1.27		
GO_CC	mitochondrion	151	19%	$1.5(10^{-10})$	$1.2(10^{-07})$	$8.5(10^{-11})$	1.65	0.54	$7.7(10^{-10})$
	except mitochondrial inner membrane	92				$1.3(10^{-04})$	1.45		
	except mitochondrial membrane part	117				$9.6(10^{-06})$	1.46		
	except mitochondrial small ribosomal subunit	143				$3.0(10^{-09})$	1.60		
GO_CC	neuron projection	33	39%	$2.3(10^{-10})$	$1.8(10^{-07})$	$4.7(10^{-11})$	3.41	0.40	$1.0(10^{-02})$
	except dendrite	16				$1.1(10^{-05})$	3.23		
	except axon	19				$2.4(10^{-07})$	3.59		
GO_CC	synapse	47	29%	$1.8(10^{-09})$	$1.4(10^{-06})$	$5.7(10^{-10})$	2.55	0.42	$6.4(10^{-04})$
	except postsynaptic membrane	28				$3.7(10^{-07})$	2.73		
GO_CC	mitochondrial membrane part	34	34%	$8.7(10^{-09})$	$6.9(10^{-06})$	$2.2(10^{-09})$	2.95	0.14	$1.8(10^{-05})$
	except mitochondrial respiratory chain	12				$4.5(10^{-04})$	2.90		
	except mitochondrial proton-transporting ATP synthase complex	28				$2.8(10^{-07})$	2.77		
GO_CC	mitochondrial inner membrane	59	24%	$3.1(10^{-08})$	$2.5(10^{-05})$	$1.3(10^{-08})$	2.11	0.45	$5.4(10^{-05})$
	except mitochondrial respiratory chain	37				$2.6(10^{-04})$	1.80		
	except mitochondrial proton-transporting ATP synthase complex	53				$4.9(10^{-07})$	1.99		
GO_CC	mitochondrial respiratory chain	22	34%	$4.9(10^{-06})$	$3.9(10^{-03})$	$1.2(10^{-06})$	2.99	0.11	$4.3(10^{-04})$
	except mitochondrial respiratory chain complex I	8				$3.0(10^{-03})$	3.02		
GO_CC	dendrite	17	41%	$5.2(10^{-06})$	$4.2(10^{-03})$	$9.8(10^{-07})$	3.60	0.17	$1.1(10^{-02})$
GO_CC	axon	14	37%	$2.0(10^{-04})$	$1.4(10^{-01})$	$4.4(10^{-05})$	3.20	0.53	$1.7(10^{-01})$
GO_CC	mitochondrial respiratory chain complex I	14	34%	$4.6(10^{-04})$	$3.0(10^{-01})$	$1.2(10^{-04})$	2.97	0.00	$1.4(10^{-03})$
GO_CC	cell junction	50	19%	$5.6(10^{-04})$	$3.6(10^{-01})$	$3.1(10^{-04})$	1.63	0.79	$8.0(10^{-02})$
GO_CC	synaptic vesicle	15	32%	$5.7(10^{-04})$	$3.6(10^{-01})$	$1.6(10^{-04})$	2.77	0.72	$2.9(10^{-01})$
GO_CC	postsynaptic membrane	19	27%	$8.7(10^{-04})$	$5.0(10^{-01})$	$3.0(10^{-04})$	2.33	0.38	$1.5(10^{-02})$
GO_CC	mitochondrial small ribosomal subunit	8	47%	$1.8(10^{-03})$	$7.6(10^{-01})$	$2.8(10^{-04})$	4.09	0.00	$6.6(10^{-02})$
GO_CC	proton-transporting two-sector ATPase complex, catalytic domain	6	60%	$3.1(10^{-03})$	$9.1(10^{-01})$	$3.2(10^{-04})$	5.21	0.68	$5.5(10^{-01})$
GO_CC	proteasome core complex (sensu Eukaryota)	8	42%	$3.8(10^{-03})$	$9.5(10^{-01})$	$7.1(10^{-04})$	3.66	0.00	$4.8(10^{-02})$
GO_CC	mitochondrial proton-transporting ATP synthase complex	6	50%	$7.9(10^{-03})$	$1.0(10^{-00})$	$1.1(10^{-03})$	4.35	0.00	$1.5(10^{-01})$
GO_CC	cytoplasm	659	14%	$5.7(10^{-13})$	$4.5(10^{-10})$	$4.6(10^{-13})$	1.21	0.88	$1.6(10^{-06})$
	except cytoplasmic part	238				$8.0(10^{-02})$	1.08		
GO_CC	mitochondrial part	96	21%	$2.6(10^{-09})$	$2.1(10^{-06})$	$1.3(10^{-09})$	1.83	0.46	$7.0(10^{-08})$
	except mitochondrial inner membrane	37				$6.1(10^{-03})$	1.52		
	except mitochondrial membrane part	62				$4.6(10^{-04})$	1.52		
	except mitochondrial small ribosomal subunit	88				$7.3(10^{-08})$	1.75		
GO_CC	mitochondrial membrane	68	22%	$2.4(10^{-07})$	$1.9(10^{-04})$	$1.1(10^{-07})$	1.89	0.54	$1.7(10^{-04})$
	except mitochondrial inner membrane	9				$4.2(10^{-01})$	1.12		
	except mitochondrial membrane part	34				$2.9(10^{-02})$	1.39		
GO_CC	organelle inner membrane	59	23%	$4.3(10^{-07})$	$3.4(10^{-04})$	$1.9(10^{-07})$	1.96	0.52	$3.1(10^{-04})$
	except mitochondrial inner membrane	0				$1.0(10^{-00})$	0.00		
GO_CC	mitochondrial envelope	69	21%	$4.8(10^{-07})$	$3.8(10^{-04})$	$2.3(10^{-07})$	1.84	0.52	$6.1(10^{-05})$
	except mitochondrial inner membrane	10				$4.7(10^{-01})$	1.06		
	except mitochondrial membrane part	35				$3.8(10^{-02})$	1.35		
GO_CC	organelle membrane	172	15%	$2.0(10^{-05})$	$1.6(10^{-02})$	$1.5(10^{-05})$	1.34	0.76	$1.6(10^{-04})$
	except mitochondrial inner membrane	113				$8.7(10^{-02})$	1.13		
	except mitochondrial membrane part	138				$1.7(10^{-02})$	1.18		
GO_CC	membrane	542	13%	$2.8(10^{-05})$	$2.2(10^{-02})$	$2.4(10^{-05})$	1.13	0.90	$3.1(10^{-04})$

	except mitochondrial inner membrane	483				$1.8(10^{-02})$	1.07		
	except mitochondrial membrane part	508				$4.2(10^{-03})$	1.09		
	except cell junction	492				$2.1(10^{-03})$	1.10		
	except postsynaptic membrane	523				$3.6(10^{-04})$	1.11		
	except proton-transporting two-sector ATPase complex, catalytic domain	536				$8.5(10^{-05})$	1.12		
GO_CC	mitochondrial ribosome	16	33%	$2.6(10^{-04})$	$1.9(10^{-01})$	$7.1(10^{-05})$	2.84	0.14	$3.7(10^{-03})$
	except mitochondrial small ribosomal subunit	8				$2.5(10^{-02})$	2.17		
GO_CC	organellar ribosome	16	33%	$2.6(10^{-04})$	$1.9(10^{-01})$	$7.1(10^{-05})$	2.84	0.14	$3.7(10^{-03})$
	except mitochondrial small ribosomal subunit	8				$2.5(10^{-02})$	2.17		
GO_CC	organelle envelope	80	17%	$3.0(10^{-04})$	$2.1(10^{-01})$	$1.9(10^{-04})$	1.48	0.76	$1.4(10^{-02})$
	except mitochondrial inner membrane	21				$8.9(10^{-01})$	0.80		
	except mitochondrial membrane part	46				$3.2(10^{-01})$	1.08		
GO_CC	envelope	80	17%	$3.2(10^{-04})$	$2.2(10^{-01})$	$2.0(10^{-04})$	1.47	0.76	$1.4(10^{-02})$
	except mitochondrial inner membrane	21				$8.9(10^{-01})$	0.80		
	except mitochondrial membrane part	46				$3.2(10^{-01})$	1.07		
GO_CC	membrane part	441	13%	$3.7(10^{-04})$	$2.5(10^{-01})$	$3.1(10^{-04})$	1.13	0.87	$4.6(10^{-05})$
	except mitochondrial membrane part	407				$2.6(10^{-02})$	1.08		
	except cell junction	391				$1.4(10^{-02})$	1.09		
	except proton-transporting two-sector ATPase complex, catalytic domain	435				$9.5(10^{-04})$	1.12		
GO_CC	clathrin-coated vesicle	23	26%	$4.2(10^{-04})$	$2.8(10^{-01})$	$1.6(10^{-04})$	2.22	1.05	$6.5(10^{-01})$
	except synaptic vesicle	8				$1.1(10^{-01})$	1.62		
GO_CC	respiratory chain complex I	14	34%	$4.6(10^{-04})$	$3.0(10^{-01})$	$1.2(10^{-04})$	2.97	0.00	$1.4(10^{-03})$
	except mitochondrial respiratory chain complex I	0				$1.0(10^{-00})$	1.00		
GO_CC	NADH dehydrogenase complex (quinone)	14	34%	$4.6(10^{-04})$	$3.0(10^{-01})$	$1.2(10^{-04})$	2.97	0.00	$1.4(10^{-03})$
	except mitochondrial respiratory chain complex I	0				$1.0(10^{-00})$	1.00		
GO_CC	membrane-bound vesicle	50	19%	$5.2(10^{-04})$	$3.4(10^{-01})$	$2.8(10^{-04})$	1.63	0.81	$1.2(10^{-01})$
	except synaptic vesicle	35				$2.7(10^{-02})$	1.39		
GO_CC	cytoplasmic membrane-bound vesicle	49	19%	$6.1(10^{-04})$	$3.8(10^{-01})$	$3.3(10^{-04})$	1.63	0.80	$1.0(10^{-01})$
	except synaptic vesicle	34				$3.1(10^{-02})$	1.38		
GO_CC	proton-transporting ATP synthase complex	10	43%	$6.2(10^{-04})$	$3.9(10^{-01})$	$1.1(10^{-04})$	3.78	0.29	$1.3(10^{-01})$
	except mitochondrial proton-transporting ATP synthase complex	4				$3.0(10^{-02})$	3.16		
GO_CC	vesicle	58	18%	$6.2(10^{-04})$	$3.9(10^{-01})$	$3.7(10^{-04})$	1.56	0.82	$9.0(10^{-02})$
	except synaptic vesicle	43				$2.5(10^{-02})$	1.35		
GO_CC	cytoplasmic vesicle	57	18%	$6.3(10^{-04})$	$3.9(10^{-01})$	$3.6(10^{-04})$	1.56	0.81	$8.8(10^{-02})$
	except synaptic vesicle	42				$2.5(10^{-02})$	1.35		
GO_CC	synapse part	21	26%	$7.8(10^{-04})$	$4.6(10^{-01})$	$2.9(10^{-04})$	2.23	0.41	$1.3(10^{-02})$
	except postsynaptic membrane	2				$3.7(10^{-01})$	1.58		
GO_CC	cell projection	47	19%	$9.2(10^{-04})$	$5.2(10^{-01})$	$5.1(10^{-04})$	1.62	1.02	$6.0(10^{-01})$
	except neuron projection	14				$9.3(10^{-01})$	0.72		
GO_CC	organellar small ribosomal subunit	8	47%	$1.8(10^{-03})$	$7.6(10^{-01})$	$2.8(10^{-04})$	4.09	0.00	$6.6(10^{-02})$
	except mitochondrial small ribosomal subunit	0				$1.0(10^{-00})$	1.00		
GO_CC	mitochondrial lumen	32	20%	$1.9(10^{-03})$	$7.8(10^{-01})$	$9.5(10^{-04})$	1.76	0.43	$7.8(10^{-04})$
	except mitochondrial small ribosomal subunit	24				$3.1(10^{-02})$	1.48		
GO_CC	mitochondrial matrix	32	20%	$1.9(10^{-03})$	$7.8(10^{-01})$	$9.5(10^{-04})$	1.76	0.43	$7.8(10^{-04})$
	except mitochondrial small ribosomal subunit	24				$3.1(10^{-02})$	1.48		
GO_CC	protein complex	196	14%	$1.9(10^{-03})$	$7.8(10^{-01})$	$1.5(10^{-03})$	1.21	0.90	$4.1(10^{-02})$
	except mitochondrial respiratory chain complex I	182				$1.4(10^{-02})$	1.16		
	except mitochondrial small ribosomal subunit	188				$6.9(10^{-03})$	1.18		
	except proteasome core complex (sensu Eukaryota)	188				$6.5(10^{-03})$	1.18		
	except proton-transporting two-sector ATPase complex, catalytic domain	190				$5.2(10^{-03})$	1.18		
	except mitochondrial proton-transporting ATP synthase complex	190				$4.9(10^{-03})$	1.18		
GO_CC	macromolecular complex	236	13%	$3.4(10^{-03})$	$9.3(10^{-01})$	$2.8(10^{-03})$	1.17	0.89	$2.1(10^{-02})$
	except mitochondrial respiratory chain complex I	222				$2.0(10^{-02})$	1.13		
	except mitochondrial small ribosomal subunit	228				$1.1(10^{-02})$	1.14		
	except proteasome core complex (sensu Eukaryota)	228				$1.0(10^{-02})$	1.14		
	except proton-transporting two-sector ATPase complex, catalytic domain	230				$8.5(10^{-03})$	1.15		
	except mitochondrial proton-transporting ATP synthase complex	230				$8.0(10^{-03})$	1.15		
GO_CC	proton-transporting two-sector ATPase complex	13	29%	$3.9(10^{-03})$	$9.5(10^{-01})$	$1.2(10^{-03})$	2.51	0.30	$2.9(10^{-02})$
	except proton-transporting two-sector ATPase complex, catalytic domain	7				$1.0(10^{-01})$	1.74		
	except mitochondrial proton-transporting ATP synthase complex	7				$7.8(10^{-02})$	1.84		
GO_CC	proteasome complex (sensu Eukaryota)	13	28%	$4.7(10^{-03})$	$9.8(10^{-01})$	$1.5(10^{-03})$	2.46	0.00	$6.3(10^{-04})$
	except proteasome core complex (sensu Eukaryota)	5				$1.9(10^{-01})$	1.61		
GO_CC	cell part	1090	12%	$5.3(10^{-03})$	$9.9(10^{-01})$	$5.3(10^{-03})$	1.01	1.00	$6.5(10^{-01})$
	except cytoplasmic part	669				$1.0(10^{-00})$	0.89		
	except neuron projection	1057				$9.5(10^{-01})$	0.99		
	except cell junction	1040				$8.1(10^{-01})$	0.99		
	except postsynaptic membrane	1071				$3.7(10^{-01})$	1.00		
	except proton-transporting two-sector ATPase complex, catalytic domain	1084				$6.4(10^{-02})$	1.01		
GO_CC	cell	1090	12%	$5.6(10^{-03})$	$9.9(10^{-01})$	$5.6(10^{-03})$	1.01	1.00	$6.5(10^{-01})$
	except cytoplasmic part	669				$1.0(10^{-00})$	0.89		
	except neuron projection	1057				$9.5(10^{-01})$	0.99		
	except cell junction	1040				$8.1(10^{-01})$	0.99		
	except postsynaptic membrane	1071				$3.7(10^{-01})$	1.00		

	except proton-transporting two-sector ATPase complex, catalytic domain	1084				$6.6(10^{-02})$	1.01		
GO_CC	coated vesicle	23	21%	$6.4(10^{-03})$	$9.9(10^{-01})$	$3.1(10^{-03})$	1.82	0.99	$5.4(10^{-01})$
	except synaptic vesicle	8				$4.4(10^{-01})$	1.10		
GO_MF	hydrogen ion transmembrane transporter activity	28	30%	$1.8(10^{-06})$	$4.7(10^{-03})$	$5.3(10^{-07})$	2.70	0.58	$5.1(10^{-02})$
	except hydrogen ion transporting ATP synthase activity, rotational mechanism	15				$1.0(10^{-03})$	2.37		
	except hydrogen ion transporting ATPase activity, rotational mechanism	15				$8.5(10^{-04})$	2.42		
GO_MF	GTPase activity	35	21%	$2.6(10^{-04})$	$4.9(10^{-01})$	$1.2(10^{-04})$	1.90	0.69	$5.5(10^{-02})$
GO_MF	hydrogen ion transporting ATP synthase activity, rotational mechanism	13	36%	$3.8(10^{-04})$	$6.2(10^{-01})$	$8.6(10^{-05})$	3.20	0.37	$7.6(10^{-02})$
GO_MF	hydrogen ion transporting ATPase activity, rotational mechanism	13	35%	$5.0(10^{-04})$	$7.2(10^{-01})$	$1.2(10^{-04})$	3.11	0.36	$6.8(10^{-02})$
GO_MF	NADH dehydrogenase (ubiquinone) activity	14	31%	$1.0(10^{-03})$	$9.3(10^{-01})$	$2.9(10^{-04})$	2.76	0.00	$6.3(10^{-04})$
GO_MF	calmodulin binding	23	22%	$3.2(10^{-03})$	$1.0(10^{-00})$	$1.4(10^{-03})$	1.92	0.69	$1.1(10^{-01})$
GO_MF	threonine endopeptidase activity	8	42%	$3.4(10^{-03})$	$1.0(10^{-00})$	$6.2(10^{-04})$	3.73	0.00	$4.5(10^{-02})$
GO_MF	phosphatase inhibitor activity	8	40%	$4.7(10^{-03})$	$1.0(10^{-00})$	$9.3(10^{-04})$	3.54	0.66	$4.0(10^{-01})$
GO_MF	GTP binding	47	16%	$8.0(10^{-03})$	$1.0(10^{-00})$	$5.0(10^{-03})$	1.46	0.70	$1.4(10^{-02})$
GO_MF	monovalent inorganic cation transmembrane transporter activity	28	28%	$1.0(10^{-05})$	$2.6(10^{-02})$	$3.4(10^{-06})$	2.48	0.53	$2.6(10^{-02})$
	except hydrogen ion transmembrane transporter activity	0				$1.0(10^{-00})$	0.00		
GO_MF	inorganic cation transmembrane transporter activity	32	25%	$3.0(10^{-05})$	$7.5(10^{-02})$	$1.2(10^{-05})$	2.20	0.57	$1.9(10^{-02})$
	except hydrogen ion transmembrane transporter activity	4				$6.1(10^{-01})$	0.96		
GO_MF	cation-transporting ATPase activity	15	33%	$2.8(10^{-04})$	$5.2(10^{-01})$	$7.3(10^{-05})$	2.95	0.44	$7.7(10^{-02})$
	except hydrogen ion transporting ATPase activity, rotational mechanism	2				$2.3(10^{-01})$	2.21		
GO_MF	ion transmembrane transporter activity	75	17%	$5.5(10^{-04})$	$7.5(10^{-01})$	$3.4(10^{-04})$	1.47	0.81	$4.3(10^{-02})$
	except hydrogen ion transmembrane transporter activity	47				$1.6(10^{-01})$	1.16		
GO_MF	oxidoreductase activity, acting on NADH or NADPH	19	27%	$6.9(10^{-04})$	$8.3(10^{-01})$	$2.4(10^{-04})$	2.37	0.28	$3.7(10^{-03})$
	except NADH dehydrogenase (ubiquinone) activity	5				$1.6(10^{-01})$	1.70		
GO_MF	substrate-specific transmembrane transporter activity	86	16%	$7.8(10^{-04})$	$8.6(10^{-01})$	$5.1(10^{-04})$	1.41	0.81	$3.1(10^{-02})$
	except hydrogen ion transmembrane transporter activity	58				$1.5(10^{-01})$	1.15		
GO_MF	oxidoreductase activity, acting on NADH or NADPH, quinone or similar compound as acceptor	15	30%	$9.3(10^{-04})$	$9.1(10^{-01})$	$2.8(10^{-04})$	2.66	0.13	$2.8(10^{-03})$
	except NADH dehydrogenase (ubiquinone) activity	1				$4.5(10^{-01})$	1.77		
GO_MF	NADH dehydrogenase (quinone) activity	14	31%	$1.0(10^{-03})$	$9.3(10^{-01})$	$2.9(10^{-04})$	2.76	0.00	$6.3(10^{-04})$
	except NADH dehydrogenase (ubiquinone) activity	0				$1.0(10^{-00})$	1.00		
GO_MF	NADH dehydrogenase activity	14	31%	$1.0(10^{-03})$	$9.3(10^{-01})$	$2.9(10^{-04})$	2.76	0.00	$6.3(10^{-04})$
	except NADH dehydrogenase (ubiquinone) activity	0				$1.0(10^{-00})$	1.00		
GO_MF	protein phosphatase regulator activity	12	32%	$2.4(10^{-03})$	$1.0(10^{-00})$	$6.6(10^{-04})$	2.80	0.52	$1.6(10^{-01})$
	except phosphatase inhibitor activity	4				$1.4(10^{-01})$	1.97		
GO_MF	cation transmembrane transporter activity	58	17%	$2.6(10^{-03})$	$1.0(10^{-00})$	$1.6(10^{-03})$	1.47	0.70	$8.2(10^{-03})$
	except hydrogen ion transmembrane transporter activity	30				$4.6(10^{-01})$	1.03		
GO_MF	catalytic activity	478	12%	$3.0(10^{-03})$	$1.0(10^{-00})$	$2.7(10^{-03})$	1.10	0.93	$1.0(10^{-02})$
	except GTPase activity	443				$4.2(10^{-02})$	1.06		
	except NADH dehydrogenase (ubiquinone) activity	464				$1.3(10^{-02})$	1.08		
	except hydrogen ion transporting ATPase activity, rotational mechanism	465				$1.3(10^{-02})$	1.08		
	except threonine endopeptidase activity	470				$7.9(10^{-03})$	1.09		
GO_MF	phosphatase regulator activity	12	31%	$3.0(10^{-03})$	$1.0(10^{-00})$	$8.6(10^{-04})$	2.73	0.51	$1.4(10^{-01})$
	except phosphatase inhibitor activity	4				$1.6(10^{-01})$	1.86		
GO_MF	substrate-specific transporter activity	102	15%	$3.1(10^{-03})$	$1.0(10^{-00})$	$2.3(10^{-03})$	1.31	0.78	$5.3(10^{-03})$
	except hydrogen ion transmembrane transporter activity	74				$2.2(10^{-01})$	1.09		
GO_MF	electron carrier activity	32	19%	$4.1(10^{-03})$	$1.0(10^{-00})$	$2.2(10^{-03})$	1.68	0.39	$1.8(10^{-04})$
	except NADH dehydrogenase (ubiquinone) activity	18				$1.6(10^{-01})$	1.29		
GO_MF	pyrophosphatase activity	73	15%	$8.4(10^{-03})$	$1.0(10^{-00})$	$5.8(10^{-03})$	1.33	0.96	$3.8(10^{-01})$
	except GTPase activity	38				$4.1(10^{-01})$	1.05		
	except hydrogen ion transporting ATPase activity, rotational mechanism	60				$8.9(10^{-02})$	1.19		
GO_MF	guanyl ribonucleotide binding	47	16%	$8.5(10^{-03})$	$1.0(10^{-00})$	$5.4(10^{-03})$	1.45	0.69	$1.4(10^{-02})$
	except GTP binding	0				$1.0(10^{-00})$	0.00		
GO_MF	guanyl nucleotide binding	47	16%	$8.5(10^{-03})$	$1.0(10^{-00})$	$5.4(10^{-03})$	1.45	0.69	$1.4(10^{-02})$
	except GTP binding	0				$1.0(10^{-00})$	0.00		
GO_MF	hydrolase activity, acting on acid anhydrides, in phosphorus-containing anhydrides	73	15%	$8.9(10^{-03})$	$1.0(10^{-00})$	$6.1(10^{-03})$	1.33	0.96	$3.7(10^{-01})$
	except GTPase activity	38				$4.2(10^{-01})$	1.04		
	except hydrogen ion transporting ATPase activity, rotational mechanism	60				$9.1(10^{-02})$	1.18		
GO_MF	hydrolase activity, acting on acid anhydrides	73	15%	$9.6(10^{-03})$	$1.0(10^{-00})$	$6.7(10^{-03})$	1.33	0.95	$3.5(10^{-01})$
	except GTPase activity	38				$4.3(10^{-01})$	1.04		
	except hydrogen ion transporting ATPase activity, rotational mechanism	60				$9.7(10^{-02})$	1.18		
GO_MF	transmembrane transporter activity	87	15%	$9.8(10^{-03})$	$1.0(10^{-00})$	$7.3(10^{-03})$	1.29	0.85	$6.5(10^{-02})$
	except hydrogen ion transmembrane transporter activity	59				$4.2(10^{-01})$	1.03		
KEGG	Oxidative phosphorylation	41	35%	$1.9(10^{-09})$	$3.8(10^{-07})$	$5.3(10^{-10})$	2.66	0.25	$1.4(10^{-04})$
KEGG	Gap junction	20	27%	$2.2(10^{-03})$	$3.6(10^{-01})$	$8.9(10^{-04})$	2.06	0.79	$2.9(10^{-01})$

Overall set of over-represented terms (PValue < 0.01; 5th column) are listed here. Every term is followed by the complementary terms of final descendants or ones inbetween which are enriched even excluding genes in descendants. The over-represented terms for themselves are listed first. [a]The proportion of genes within group in total 13,216 genes which were annotated with the specific term [b]EASE score [c]Hypergeometric test for overrepresentation [d]Fold enrichment of the term in the gene group [e]Fold enrichment of the term in the opposite gene group (Table S2, genes expression of which increases as age) [f]Hypergeometric test for underrepresentation of genes in the opposite group
