# Supplementary material for: Transcriptome-Wide Assessment of Human Brain and Lymphocyte Senescence
Source: PLoS One. 2008 Aug 20;3(8):e3024. doi: 10.1371/journal.pone.0003024 (PMC2515343; doi:10.1371/journal.pone.0003024)
Supplement: Table S2 — Terms in the Gene Ontology and KEGG pathway databases enriched among genes that increased expression with advancing age in brain (1943 genes in total 13,216) (0.09 MB PDF) [file pone.0003024.s003.pdf]

Table S2. Terms in the Gene Ontology and KEGG pathway databases enriched among genes that increased expression with advancing age in brain (1943 genes in total 13,216)

Category	Term	Count	%Hit[a]	PValue[b]	Bonferroni	HGeom[c]	Fold[d]	Fold-U[e]	HGe-U[f]
GO_BP	regulation of transcription, DNA-dependent	323	21%	$2.1(10^{-13})$	$1.0(10^{-09})$	$1.3(10^{-13})$	1.43	0.56	$6.1(10^{-13})$
	exceptnegative regulation of transcription from RNA polymerase II promoter	294				$3.7(10^{-11})$	1.40		
GO_BP	chromosome organization and biogenesis	77	27%	$1.4(10^{-07})$	$6.8(10^{-04})$	$6.7(10^{-08})$	1.80	0.53	$1.4(10^{-03})$
	exceptchromatin modification	29				$2.3(10^{-03})$	1.70		
	exceptchromatin assembly or disassembly	53				$5.3(10^{-06})$	1.82		
GO_BP	DNA packaging	62	27%	$4.7(10^{-06})$	$2.2(10^{-02})$	$2.2(10^{-06})$	1.78	0.49	$1.9(10^{-03})$
GO_BP	DNA metabolic process	127	22%	$6.4(10^{-06})$	$3.0(10^{-02})$	$3.9(10^{-06})$	1.45	0.59	$7.6(10^{-05})$
	exceptDNA replication	86				$6.1(10^{-04})$	1.39		
GO_BP	chromatin modification	48	28%	$1.5(10^{-05})$	$6.8(10^{-02})$	$6.6(10^{-06})$	1.87	0.57	$2.3(10^{-02})$
GO_BP	negative regulation of transcription from RNA polymerase II promoter	29	27%	$1.5(10^{-03})$	$1.0(10^{-00})$	$6.7(10^{-04})$	1.83	0.33	$5.4(10^{-03})$
GO_BP	DNA replication	41	24%	$2.4(10^{-03})$	$1.0(10^{-00})$	$1.3(10^{-03})$	1.59	0.36	$6.4(10^{-04})$
GO_BP	cell-matrix adhesion	18	31%	$3.8(10^{-03})$	$1.0(10^{-00})$	$1.5(10^{-03})$	2.07	0.61	$2.0(10^{-01})$
GO_BP	immune system development	33	24%	$6.5(10^{-03})$	$1.0(10^{-00})$	$3.5(10^{-03})$	1.60	0.51	$2.1(10^{-02})$
GO_BP	B cell mediated immunity	12	36%	$6.6(10^{-03})$	$1.0(10^{-00})$	$2.0(10^{-03})$	2.43	0.00	$1.9(10^{-02})$
GO_BP	chromatin assembly or disassembly	24	26%	$6.7(10^{-03})$	$1.0(10^{-00})$	$3.2(10^{-03})$	1.76	0.68	$1.8(10^{-01})$
GO_BP	chordate embryonic development	22	27%	$6.9(10^{-03})$	$1.0(10^{-00})$	$3.2(10^{-03})$	1.81	0.66	$1.8(10^{-01})$
GO_BP	leukotriene biosynthetic process	7	54%	$7.4(10^{-03})$	$1.0(10^{-00})$	$1.2(10^{-03})$	3.60	0.00	$2.1(10^{-01})$
GO_BP	regulation of transcription	340	21%	$4.2(10^{-13})$	$2.0(10^{-09})$	$2.6(10^{-13})$	1.40	0.57	$3.9(10^{-13})$
	exceptregulation of transcription, DNA-dependent	17				$4.2(10^{-01})$	1.07		
GO_BP	regulation of nucleobase, nucleoside, nucleotide and nucleic acid metabolic process	347	21%	$5.6(10^{-13})$	$2.7(10^{-09})$	$3.8(10^{-13})$	1.39	0.58	$3.6(10^{-13})$
	exceptregulation of transcription, DNA-dependent	24				$4.1(10^{-01})$	1.06		
GO_BP	RNA biosynthetic process	329	21%	$5.9(10^{-13})$	$2.8(10^{-09})$	$3.8(10^{-13})$	1.41	0.58	$4.7(10^{-12})$
	exceptregulation of transcription, DNA-dependent	6				$7.1(10^{-01})$	0.87		
GO_BP	transcription, DNA-dependent	328	21%	$7.6(10^{-13})$	$3.7(10^{-09})$	$4.9(10^{-13})$	1.41	0.58	$2.8(10^{-12})$
	exceptregulation of transcription, DNA-dependent	5				$7.9(10^{-01})$	0.78		
GO_BP	RNA metabolic process	405	20%	$9.7(10^{-13})$	$4.7(10^{-09})$	$6.5(10^{-13})$	1.34	0.63	$2.7(10^{-12})$
	exceptregulation of transcription, DNA-dependent	82				$2.1(10^{-01})$	1.09		
GO_BP	transcription	351	21%	$9.8(10^{-13})$	$4.7(10^{-09})$	$6.7(10^{-13})$	1.39	0.58	$2.1(10^{-13})$
	exceptregulation of transcription, DNA-dependent	28				$4.6(10^{-01})$	1.03		
GO_BP	regulation of metabolic process	382	20%	$4.0(10^{-12})$	$1.9(10^{-08})$	$2.8(10^{-12})$	1.35	0.66	$4.4(10^{-10})$
	exceptregulation of transcription, DNA-dependent	59				$4.2(10^{-01})$	1.03		
GO_BP	regulation of cellular metabolic process	368	20%	$6.4(10^{-12})$	$3.1(10^{-08})$	$4.3(10^{-12})$	1.35	0.62	$8.8(10^{-12})$
	exceptregulation of transcription, DNA-dependent	45				$5.6(10^{-01})$	0.99		
GO_BP	regulation of gene expression	352	20%	$1.2(10^{-11})$	$5.8(10^{-08})$	$8.4(10^{-12})$	1.36	0.60	$1.4(10^{-12})$
	exceptregulation of transcription, DNA-dependent	29				$7.8(10^{-01})$	0.89		
GO_BP	nucleobase, nucleoside, nucleotide and nucleic acid metabolic process	502	19%	$6.7(10^{-11})$	$3.2(10^{-07})$	$4.9(10^{-11})$	1.26	0.70	$1.7(10^{-11})$
	exceptregulation of transcription, DNA-dependent	179				$3.2(10^{-01})$	1.03		
	exceptDNA metabolic process	375				$8.8(10^{-06})$	1.20		
GO_BP	gene expression	459	19%	$2.6(10^{-10})$	$1.3(10^{-06})$	$2.0(10^{-10})$	1.27	0.69	$4.6(10^{-11})$
	exceptregulation of transcription, DNA-dependent	136				$5.3(10^{-01})$	1.00		
GO_BP	regulation of cellular process	531	18%	$1.0(10^{-08})$	$4.8(10^{-05})$	$8.0(10^{-09})$	1.21	0.82	$9.1(10^{-06})$
	exceptregulation of transcription, DNA-dependent	208				$6.8(10^{-01})$	0.98		
GO_BP	regulation of biological process	558	18%	$1.7(10^{-07})$	$8.0(10^{-04})$	$1.3(10^{-07})$	1.18	0.84	$5.3(10^{-05})$
	exceptregulation of transcription, DNA-dependent	235				$8.3(10^{-01})$	0.95		
GO_BP	biopolymer metabolic process	623	17%	$2.4(10^{-07})$	$1.2(10^{-03})$	$2.0(10^{-07})$	1.16	0.79	$4.8(10^{-09})$
	exceptregulation of transcription, DNA-dependent	300				$8.0(10^{-01})$	0.96		
	exceptDNA metabolic process	496				$2.5(10^{-03})$	1.10		
GO_BP	establishment and/or maintenance of chromatin architecture	62	27%	$3.5(10^{-06})$	$1.6(10^{-02})$	$1.6(10^{-06})$	1.79	0.50	$2.2(10^{-03})$
	exceptchromatin modification	14				$5.7(10^{-02})$	1.56		
	exceptchromatin assembly or disassembly	38				$1.3(10^{-04})$	1.81		
GO_BP	macromolecule metabolic process	785	17%	$5.3(10^{-06})$	$2.5(10^{-02})$	$4.7(10^{-06})$	1.11	0.86	$4.9(10^{-07})$
	exceptregulation of transcription, DNA-dependent	462				$9.1(10^{-01})$	0.96		
	exceptDNA metabolic process	658				$1.8(10^{-02})$	1.06		
GO_BP	biological regulation	589	17%	$9.3(10^{-06})$	$4.4(10^{-02})$	$7.8(10^{-06})$	1.14	0.85	$3.4(10^{-05})$
	exceptregulation of transcription, DNA-dependent	266				$9.6(10^{-01})$	0.92		
GO_BP	negative regulation of metabolic process	75	23%	$1.7(10^{-04})$	$5.6(10^{-01})$	$1.0(10^{-04})$	1.51	0.67	$1.5(10^{-02})$
	exceptnegative regulation of transcription from RNA polymerase II	46				$1.5(10^{-02})$	1.37		

	promoter								
GO_BP	primary metabolic process	875	16%	$1.8(10^{-04})$	$5.7(10^{-01})$	$1.7(10^{-04})$	1.07	0.89	$2.9(10^{-06})$
	exceptregulation of transcription, DNA-dependent	552				$9.9(10^{-01})$	0.94		
	exceptDNA metabolic process	748				$1.2(10^{-01})$	1.03		
	exceptleukotriene biosynthetic process	868				$5.3(10^{-04})$	1.07		
GO_BP	negative regulation of transcription, DNA-dependent	40	25%	$8.4(10^{-04})$	$9.8(10^{-01})$	$4.3(10^{-04})$	1.69	0.56	$2.5(10^{-02})$
	exceptnegative regulation of transcription from RNA polymerase II promoter	11				$1.5(10^{-01})$	1.41		
GO_BP	negative regulation of cellular metabolic process	64	22%	$1.5(10^{-03})$	$1.0(10^{-00})$	$9.0(10^{-04})$	1.46	0.57	$3.5(10^{-03})$
	exceptnegative regulation of transcription from RNA polymerase II promoter	35				$9.2(10^{-02})$	1.25		
GO_BP	cellular metabolic process	861	16%	$1.5(10^{-03})$	$1.0(10^{-00})$	$1.4(10^{-03})$	1.06	0.90	$2.6(10^{-05})$
	exceptregulation of transcription, DNA-dependent	538				$1.0(10^{-00})$	0.92		
	exceptDNA metabolic process	734				$2.8(10^{-01})$	1.01		
	exceptleukotriene biosynthetic process	854				$3.7(10^{-03})$	1.06		
GO_BP	immune effector process	21	31%	$1.7(10^{-03})$	$1.0(10^{-00})$	$6.6(10^{-04})$	2.06	0.13	$2.7(10^{-03})$
	exceptB cell mediated immunity	9				$6.8(10^{-02})$	1.72		
GO_BP	cell-substrate adhesion	19	31%	$2.7(10^{-03})$	$1.0(10^{-00})$	$1.0(10^{-03})$	2.08	0.58	$1.7(10^{-01})$
	exceptcell-matrix adhesion	1				$3.9(10^{-01})$	2.23		
GO_BP	negative regulation of transcription	50	22%	$3.6(10^{-03})$	$1.0(10^{-00})$	$2.2(10^{-03})$	1.48	0.55	$6.8(10^{-03})$
	exceptnegative regulation of transcription from RNA polymerase II promoter	21				$2.4(10^{-01})$	1.18		
GO_BP	negative regulation of biological process	158	18%	$4.2(10^{-03})$	$1.0(10^{-00})$	$3.2(10^{-03})$	1.22	0.92	$2.1(10^{-01})$
	exceptnegative regulation of transcription from RNA polymerase II promoter	129				$6.1(10^{-02})$	1.13		
GO_BP	negative regulation of nucleobase, nucleoside, nucleotide and nucleic acid metabolic process	53	22%	$4.4(10^{-03})$	$1.0(10^{-00})$	$2.7(10^{-03})$	1.45	0.55	$4.3(10^{-03})$
	exceptnegative regulation of transcription from RNA polymerase II promoter	24				$2.4(10^{-01})$	1.16		
GO_BP	negative regulation of cellular process	153	18%	$4.4(10^{-03})$	$1.0(10^{-00})$	$3.4(10^{-03})$	1.22	0.91	$1.8(10^{-01})$
	exceptnegative regulation of transcription from RNA polymerase II promoter	124				$6.6(10^{-02})$	1.13		
GO_BP	embryonic development ending in birth or egg hatching	22	27%	$6.9(10^{-03})$	$1.0(10^{-00})$	$3.2(10^{-03})$	1.81	0.66	$1.8(10^{-01})$
	exceptchordate embryonic development	0				$1.0(10^{-00})$	1.00		
GO_BP	metabolic process	930	16%	$7.0(10^{-03})$	$1.0(10^{-00})$	$6.6(10^{-03})$	1.05	0.92	$2.4(10^{-04})$
	exceptregulation of transcription, DNA-dependent	607				$1.0(10^{-00})$	0.92		
	exceptDNA metabolic process	803				$4.9(10^{-01})$	1.00		
	exceptleukotriene biosynthetic process	923				$1.5(10^{-02})$	1.04		
GO_BP	alkene biosynthetic process	7	54%	$7.4(10^{-03})$	$1.0(10^{-00})$	$1.2(10^{-03})$	3.60	0.00	$2.1(10^{-01})$
	exceptleukotriene biosynthetic process	0				$1.0(10^{-00})$	1.00		
GO_CC	nucleus	609	19%	$1.2(10^{-13})$	$9.6(10^{-11})$	$9.0(10^{-14})$	1.26	0.75	$3.7(10^{-11})$
	exceptnuclear speck	587				$8.4(10^{-12})$	1.24		
	excepthistone deacetylase complex	599				$1.4(10^{-12})$	1.24		
GO_CC	chromosomal part	48	23%	$1.5(10^{-03})$	$7.0(10^{-01})$	$8.4(10^{-04})$	1.56	0.54	$7.3(10^{-03})$
GO_CC	nuclear speck	22	30%	$1.5(10^{-03})$	$7.0(10^{-01})$	$5.9(10^{-04})$	2.04	0.36	$2.5(10^{-02})$
GO_CC	histone deacetylase complex	10	42%	$5.2(10^{-03})$	$9.8(10^{-01})$	$1.3(10^{-03})$	2.82	0.72	$4.7(10^{-01})$
GO_CC	MHC class II protein complex	6	67%	$5.2(10^{-03})$	$9.8(10^{-01})$	$5.8(10^{-04})$	4.52	0.00	$3.3(10^{-01})$
GO_CC	MHC protein complex	11	55%	$2.1(10^{-04})$	$1.5(10^{-01})$	$3.2(10^{-05})$	3.73	0.00	$8.7(10^{-02})$
	exceptMHC class II protein complex	5				$1.5(10^{-02})$	3.08		
GO_CC	nucleoplasm part	73	21%	$1.6(10^{-03})$	$7.1(10^{-01})$	$9.9(10^{-04})$	1.42	0.62	$4.2(10^{-03})$
	exceptnuclear speck	51				$5.0(10^{-02})$	1.25		
	excepthistone deacetylase complex	63				$1.2(10^{-02})$	1.31		
GO_CC	nuclear body	27	28%	$1.6(10^{-03})$	$7.3(10^{-01})$	$7.3(10^{-04})$	1.87	0.27	$2.6(10^{-03})$
	exceptnuclear speck	5				$3.1(10^{-01})$	1.35		
GO_CC	chromosome	54	21%	$4.8(10^{-03})$	$9.8(10^{-01})$	$3.0(10^{-03})$	1.44	0.55	$3.4(10^{-03})$
	exceptchromosomal part	6				$6.9(10^{-01})$	0.88		
GO_CC	nucleoplasm	78	19%	$9.2(10^{-03})$	$1.0(10^{-00})$	$6.5(10^{-03})$	1.31	0.65	$3.8(10^{-03})$
	exceptnuclear speck	56				$1.5(10^{-01})$	1.15		
	excepthistone deacetylase complex	68				$4.9(10^{-02})$	1.21		
GO_MF	DNA binding	322	22%	$6.8(10^{-14})$	$1.7(10^{-10})$	$4.4(10^{-14})$	1.44	0.52	$1.1(10^{-14})$
	excepttranscription factor activity	205				$7.9(10^{-10})$	1.47		
GO_MF	zinc ion binding	321	21%	$1.7(10^{-11})$	$4.3(10^{-08})$	$1.1(10^{-11})$	1.39	0.64	$6.3(10^{-09})$
GO_MF	transcription regulator activity	190	21%	$1.1(10^{-06})$	$2.7(10^{-03})$	$7.5(10^{-07})$	1.38	0.65	$1.8(10^{-05})$
	excepttranscription factor activity	73				$3.7(10^{-03})$	1.35		
GO_MF	transcription factor activity	117	21%	$1.0(10^{-04})$	$2.2(10^{-01})$	$6.7(10^{-05})$	1.39	0.57	$5.3(10^{-05})$
GO_MF	calcium-dependent phospholipid binding	9	47%	$4.1(10^{-03})$	$1.0(10^{-00})$	$8.6(10^{-04})$	3.15	1.86	$9.5(10^{-01})$
GO_MF	MHC class II receptor activity	6	67%	$5.7(10^{-03})$	$1.0(10^{-00})$	$6.4(10^{-04})$	4.43	0.00	$3.4(10^{-01})$
GO_MF	nucleic acid binding	454	20%	$1.8(10^{-12})$	$4.5(10^{-09})$	$1.2(10^{-12})$	1.31	0.63	$4.8(10^{-14})$
	exceptDNA binding	132				$1.8(10^{-01})$	1.08		
GO_MF	transition metal ion binding	357	19%	$5.0(10^{-08})$	$1.3(10^{-04})$	$3.6(10^{-08})$	1.28	0.68	$1.0(10^{-08})$
	exceptzinc ion binding	36				$9.8(10^{-01})$	0.74		
GO_MF	cation binding	453	18%	$3.3(10^{-06})$	$8.3(10^{-03})$	$2.7(10^{-06})$	1.19	0.79	$4.0(10^{-06})$
	exceptzinc ion binding	132				$9.6(10^{-01})$	0.88		
GO_MF	metal ion binding	485	18%	$1.2(10^{-05})$	$3.1(10^{-02})$	$1.0(10^{-05})$	1.17	0.87	$1.6(10^{-03})$
	exceptzinc ion binding	164				$9.6(10^{-01})$	0.89		

GO_MF	ion binding	491	17%	$2.0(10^{-05})$	$4.9(10^{-02})$	$1.7(10^{-05})$	1.16	0.87	$1.9(10^{-03})$
	exceptzinc ion binding	170				$9.7(10^{-01})$	0.89		
GO_MF	binding	1303	16%	$3.8(10^{-05})$	$9.3(10^{-02})$	$3.7(10^{-05})$	1.04	0.96	$1.8(10^{-03})$
	exceptDNA binding	981				$1.0(10^{-00})$	0.95		
	exceptzinc ion binding	982				$9.9(10^{-01})$	0.96		
	exceptcalcium-dependent phospholipid binding	1294				$2.8(10^{-04})$	1.04		
KEGG	Focal adhesion	33	22%	$6.8(10^{-03})$	$7.4(10^{-01})$	$3.8(10^{-03})$	1.58	1.05	$6.6(10^{-01})$

Overall set of over-represented terms (PValue < 0.01; 5th column) are listed here. Every term is followed by the complementary terms of final descendants or ones inbetween which are enriched even excluding genes in descendants. The over-represented terms for themselves are listed first. [a]The proportion of genes within group in total 13,216 genes which were annotated with the specific term [b]EASE score [c]Hypergeometric test for overrepresentation [d]Fold enrichment of the term in the gene group [e]Fold enrichment of the term in the opposite gene group (Table S1, genes expression of which decreases as age) [f]Hypergeometric test for underrepresentation of genes in the opposite group
