# Supplementary material for: Transcriptome-Wide Assessment of Human Brain and Lymphocyte Senescence
Source: PLoS One. 2008 Aug 20;3(8):e3024. doi: 10.1371/journal.pone.0003024 (PMC2515343; doi:10.1371/journal.pone.0003024)
Supplement: Table S3 — Terms in the Gene Ontology and KEGG pathway databases enriched among genes that decreased expression with advancing age in the human lymphocytes (1878 genes in total 13,232) (0.13 MB PDF) [file pone.0003024.s004.pdf]

Table S3. Terms in the Gene Ontology and KEGG pathway databases enriched among genes that decreased expression with advancing age in the human lymphocytes (1878 genes in total 13,232)

Category	Term	Count	%Hit[a]	PValue[b]	Bonferroni	HGeom[c]	Fold[d]	Fold-U[e]	HGe-U[f]
GO_BP	translation	139	33%	$4.2(10^{-21})$	$2.0(10^{-17})$	$2.3(10^{-21})$	2.19	0.60	$8.7(10^{-04})$
	except tRNA aminoacylation for protein translation	123				$6.1(10^{-19})$	2.19		
GO_BP	cellular biosynthetic process	210	28%	$6.7(10^{-21})$	$3.2(10^{-17})$	$5.4(10^{-21})$	1.83	0.79	$1.5(10^{-02})$
	except translation	71				$1.9(10^{-03})$	1.39		
GO_BP	gene expression	501	21%	$4.0(10^{-18})$	$1.9(10^{-14})$	$6.5(10^{-18})$	1.36	0.70	$3.1(10^{-11})$
	except translation	362				$4.7(10^{-05})$	1.19		
	except RNA processing	404				$3.6(10^{-11})$	1.31		
GO_BP	ribosome biogenesis and assembly	32	44%	$1.5(10^{-08})$	$7.2(10^{-05})$	$3.5(10^{-09})$	2.90	0.23	$5.9(10^{-03})$
	except rRNA processing	10				$4.3(10^{-04})$	3.15		
GO_BP	tRNA metabolic process	38	39%	$2.9(10^{-08})$	$1.4(10^{-04})$	$8.1(10^{-09})$	2.57	0.52	$4.7(10^{-02})$
	except tRNA processing	15				$1.4(10^{-03})$	2.26		
	except tRNA aminoacylation for protein translation	22				$9.2(10^{-07})$	2.91		
GO_BP	RNA metabolic process	382	19%	$2.2(10^{-07})$	$1.0(10^{-03})$	$1.6(10^{-07})$	1.24	0.71	$6.9(10^{-09})$
	except RNA processing	285				$2.7(10^{-03})$	1.15		
	except tRNA metabolic process	344				$1.7(10^{-04})$	1.17		
GO_BP	RNA processing	97	24%	$8.9(10^{-07})$	$4.2(10^{-03})$	$4.8(10^{-07})$	1.61	0.23	$2.5(10^{-11})$
	except tRNA processing	74				$7.6(10^{-04})$	1.42		
	except rRNA processing	75				$5.6(10^{-04})$	1.43		
GO_BP	tRNA processing	23	43%	$4.3(10^{-06})$	$2.0(10^{-02})$	$1.1(10^{-06})$	2.82	0.63	$2.2(10^{-01})$
	except tRNA modification	15				$5.9(10^{-04})$	2.42		
GO_BP	rRNA processing	22	42%	$8.4(10^{-06})$	$3.9(10^{-02})$	$2.1(10^{-06})$	2.80	0.16	$1.1(10^{-02})$
GO_BP	tRNA modification	8	62%	$1.3(10^{-03})$	$1.0(10^{-00})$	$1.7(10^{-04})$	4.07	0.00	$1.9(10^{-01})$
GO_BP	protein import into mitochondrion	7	64%	$2.7(10^{-03})$	$1.0(10^{-00})$	$3.3(10^{-04})$	4.21	0.00	$2.5(10^{-01})$
GO_BP	tRNA aminoacylation for protein translation	16	33%	$3.6(10^{-03})$	$1.0(10^{-00})$	$1.3(10^{-03})$	2.21	0.35	$6.6(10^{-02})$
GO_BP	nucleobase, nucleoside and nucleotide metabolic process	40	23%	$6.2(10^{-03})$	$1.0(10^{-00})$	$3.6(10^{-03})$	1.52	0.78	$1.7(10^{-01})$
GO_BP	protein-RNA complex assembly	25	26%	$9.6(10^{-03})$	$1.0(10^{-00})$	$4.8(10^{-03})$	1.69	0.78	$2.6(10^{-01})$
GO_BP	macromolecule biosynthetic process	168	27%	$5.4(10^{-16})$	$2.6(10^{-12})$	$2.6(10^{-16})$	1.81	0.77	$1.7(10^{-02})$
	except translation	29				$5.4(10^{-01})$	0.99		
GO_BP	biosynthetic process	244	24%	$1.7(10^{-15})$	$8.4(10^{-12})$	$9.6(10^{-16})$	1.59	0.89	$9.9(10^{-02})$
	except cellular biosynthetic process	34				$8.3(10^{-01})$	0.88		
GO_BP	cellular metabolic process	923	17%	$2.2(10^{-14})$	$1.1(10^{-10})$	$1.8(10^{-14})$	1.15	0.87	$2.7(10^{-08})$
	except cellular biosynthetic process	713				$5.4(10^{-02})$	1.04		
	except RNA metabolic process	541				$1.5(10^{-03})$	1.10		
	except nucleobase, nucleoside and nucleotide metabolic process	883				$1.5(10^{-11})$	1.14		
GO_BP	metabolic process	986	17%	$4.9(10^{-13})$	$2.3(10^{-09})$	$4.3(10^{-13})$	1.13	0.87	$1.1(10^{-09})$
	except cellular biosynthetic process	776				$1.7(10^{-01})$	1.02		
	except RNA metabolic process	604				$1.3(10^{-02})$	1.07		
	except nucleobase, nucleoside and nucleotide metabolic process	946				$3.7(10^{-10})$	1.12		
	except protein-RNA complex assembly	961				$6.6(10^{-11})$	1.12		
GO_BP	primary metabolic process	919	17%	$1.4(10^{-12})$	$6.4(10^{-09})$	$1.2(10^{-12})$	1.14	0.89	$4.4(10^{-06})$
	except translation	780				$1.2(10^{-02})$	1.05		
	except RNA metabolic process	537				$8.3(10^{-03})$	1.08		
	except nucleobase, nucleoside and nucleotide metabolic process	879				$5.6(10^{-10})$	1.13		
GO_BP	nucleobase, nucleoside, nucleotide and nucleic acid metabolic process	499	19%	$1.1(10^{-09})$	$5.2(10^{-06})$	$8.6(10^{-10})$	1.23	0.69	$2.7(10^{-13})$
	except RNA metabolic process	117				$1.2(10^{-02})$	1.21		
	except nucleobase, nucleoside and nucleotide metabolic process	459				$7.7(10^{-08})$	1.21		
GO_BP	ribonucleoprotein complex biogenesis and assembly	55	33%	$1.3(10^{-08})$	$6.3(10^{-05})$	$4.8(10^{-09})$	2.18	0.56	$1.8(10^{-02})$
	except ribosome biogenesis and assembly	23				$1.1(10^{-02})$	1.62		
	except protein-RNA complex assembly	30				$1.4(10^{-08})$	2.88		
GO_BP	macromolecule metabolic process	800	17%	$2.0(10^{-08})$	$9.3(10^{-05})$	$1.7(10^{-08})$	1.13	0.88	$1.2(10^{-05})$
	except translation	661				$1.6(10^{-01})$	1.03		
	except RNA metabolic process	418				$1.3(10^{-01})$	1.05		
	except protein-RNA complex assembly	775				$5.0(10^{-07})$	1.12		
GO_BP	rRNA metabolic process	23	42%	$6.2(10^{-06})$	$2.9(10^{-02})$	$1.6(10^{-06})$	2.77	0.31	$3.4(10^{-02})$
	except rRNA processing	1				$3.9(10^{-01})$	2.21		
GO_BP	RNA modification	15	48%	$6.3(10^{-05})$	$2.6(10^{-01})$	$1.2(10^{-05})$	3.20	0.55	$2.7(10^{-01})$
	except tRNA modification	7				$1.2(10^{-02})$	2.57		
GO_BP	amino acid metabolic process	49	24%	$7.8(10^{-04})$	$9.7(10^{-01})$	$4.3(10^{-04})$	1.60	0.92	$3.8(10^{-01})$
	except tRNA aminoacylation for protein translation	33				$2.3(10^{-02})$	1.41		
GO_BP	biopolymer metabolic process	589	17%	$1.4(10^{-03})$	$1.0(10^{-00})$	$1.3(10^{-03})$	1.09	0.81	$4.5(10^{-08})$
	except RNA metabolic process	207				$9.7(10^{-01})$	0.90		

GO_BP	carboxylic acid metabolic process	78	21%	$3.1(10^{-03})$	$1.0(10^{-00})$	$2.1(10^{-03})$	1.36	1.03	$6.2(10^{-01})$
	except tRNA aminoacylation for protein translation	62				$3.9(10^{-02})$	1.24		
GO_BP	tRNA aminoacylation	16	33%	$3.6(10^{-03})$	$1.0(10^{-00})$	$1.3(10^{-03})$	2.21	0.35	$6.6(10^{-02})$
	except tRNA aminoacylation for protein translation	0				1.0	1.00		
GO_BP	amino acid activation	16	33%	$3.6(10^{-03})$	$1.0(10^{-00})$	$1.3(10^{-03})$	2.21	0.35	$6.6(10^{-02})$
	except tRNA aminoacylation for protein translation	0				1.0	1.00		
GO_BP	organic acid metabolic process	78	20%	$3.6(10^{-03})$	$1.0(10^{-00})$	$2.4(10^{-03})$	1.36	1.04	$6.6(10^{-01})$
	except tRNA aminoacylation for protein translation	62				$4.3(10^{-02})$	1.23		
GO_BP	protein targeting to mitochondrion	9	47%	$4.2(10^{-03})$	$1.0(10^{-00})$	$8.8(10^{-04})$	3.14	0.45	$3.3(10^{-01})$
	except protein import into mitochondrion	2				$3.5(10^{-01})$	1.65		
GO_BP	amino acid and derivative metabolic process	54	22%	$4.2(10^{-03})$	$1.0(10^{-00})$	$2.6(10^{-03})$	1.45	0.86	$2.3(10^{-01})$
	except tRNA aminoacylation for protein translation	38				$7.1(10^{-02})$	1.26		
GO_CC	ribosome	86	46%	$2.0(10^{-23})$	$1.5(10^{-20})$	$5.2(10^{-24})$	3.02	0.09	$1.1(10^{-08})$
	except ribosomal subunit	26				$3.9(10^{-05})$	2.22		
GO_CC	ribosomal subunit	60	55%	$4.2(10^{-21})$	$3.3(10^{-18})$	$7.5(10^{-22})$	3.59	0.08	$1.1(10^{-05})$
	except small ribosomal subunit	30				$4.7(10^{-11})$	3.46		
	except large ribosomal subunit	29				$7.6(10^{-12})$	3.74		
GO_CC	ribonucleoprotein complex	136	33%	$1.6(10^{-20})$	$1.3(10^{-17})$	$8.8(10^{-21})$	2.19	0.22	$3.5(10^{-12})$
	except ribosome	50				$2.3(10^{-03})$	1.48		
GO_CC	organelle lumen	199	26%	$9.0(10^{-16})$	$7.0(10^{-13})$	$4.3(10^{-16})$	1.71	0.55	$2.2(10^{-07})$
	except nucleolus	147				$1.0(10^{-08})$	1.55		
	except mitochondrial matrix	148				$2.1(10^{-09})$	1.58		
	except nuclear speck	176				$2.0(10^{-13})$	1.67		
GO_CC	mitochondrion	180	25%	$5.0(10^{-12})$	$3.9(10^{-09})$	$2.7(10^{-12})$	1.62	0.60	$7.4(10^{-06})$
	except mitochondrial part	73				$2.0(10^{-04})$	1.49		
GO_CC	small ribosomal subunit	30	57%	$2.7(10^{-11})$	$2.2(10^{-08})$	$3.9(10^{-12})$	3.72	0.00	$1.1(10^{-03})$
	except mitochondrial small ribosomal subunit	19				$1.6(10^{-07})$	3.47		
GO_CC	large ribosomal subunit	31	53%	$1.4(10^{-10})$	$1.1(10^{-07})$	$2.4(10^{-11})$	3.45	0.14	$4.6(10^{-03})$
GO_CC	cytosolic part	47	39%	$3.1(10^{-10})$	$2.5(10^{-07})$	$8.6(10^{-11})$	2.60	0.63	$8.0(10^{-02})$
GO_CC	nucleolus	52	37%	$5.6(10^{-10})$	$4.4(10^{-07})$	$1.7(10^{-10})$	2.42	0.47	$8.7(10^{-03})$
	except nucleolar part	39				$8.5(10^{-08})$	2.35		
GO_CC	intracellular organelle part	477	19%	$4.9(10^{-09})$	$3.8(10^{-06})$	$3.8(10^{-09})$	1.24	0.83	$1.4(10^{-04})$
	except ribosomal subunit	417				$1.0(10^{-03})$	1.13		
	except mitochondrial part	370				$9.3(10^{-04})$	1.14		
	except nucleolus	425				$4.5(10^{-05})$	1.17		
	except nuclear speck	454				$1.8(10^{-07})$	1.21		
GO_CC	mitochondrial part	107	26%	$6.4(10^{-09})$	$5.0(10^{-06})$	$3.2(10^{-09})$	1.72	0.63	$1.9(10^{-03})$
	except mitochondrial matrix	56				$3.6(10^{-03})$	1.42		
	except mitochondrial inner membrane	60				$2.9(10^{-08})$	2.01		
GO_CC	mitochondrial matrix	51	34%	$1.5(10^{-08})$	$1.2(10^{-05})$	$5.2(10^{-09})$	2.25	0.39	$1.8(10^{-03})$
	except mitochondrial small ribosomal subunit	40				$7.6(10^{-06})$	1.99		
GO_CC	mitochondrial small ribosomal subunit	11	65%	$4.5(10^{-05})$	$3.4(10^{-02})$	$5.0(10^{-06})$	4.25	0.00	$1.1(10^{-01})$
GO_CC	nuclear speck	23	31%	$1.3(10^{-03})$	$6.5(10^{-01})$	$5.3(10^{-04})$	2.02	0.22	$4.1(10^{-03})$
GO_CC	nucleolar part	13	41%	$1.7(10^{-03})$	$7.4(10^{-01})$	$4.5(10^{-04})$	2.67	0.26	$8.8(10^{-02})$
GO_CC	mitochondrial inner membrane	47	22%	$7.4(10^{-03})$	$1.0(10^{-00})$	$4.6(10^{-03})$	1.45	0.70	$5.9(10^{-02})$
GO_CC	membrane-enclosed lumen	199	26%	$9.0(10^{-16})$	$7.0(10^{-13})$	$4.3(10^{-16})$	1.71	0.55	$2.2(10^{-07})$
	except organelle lumen	0				1.0	1.00		
GO_CC	organelle	980	17%	$3.2(10^{-13})$	$2.6(10^{-10})$	$2.8(10^{-13})$	1.14	0.89	$9.8(10^{-07})$
	except intracellular organelle part	503				$4.0(10^{-02})$	1.06		
	except organelle lumen	781				$1.5(10^{-02})$	1.05		
	except mitochondrion	800				$1.6(10^{-03})$	1.07		
	except ribosome	894				$1.2(10^{-04})$	1.08		
GO_CC	intracellular organelle	979	17%	$4.0(10^{-13})$	$3.2(10^{-10})$	$3.4(10^{-13})$	1.14	0.89	$1.2(10^{-06})$
	except intracellular organelle part	502				$4.2(10^{-02})$	1.06		
	except mitochondrion	799				$1.7(10^{-03})$	1.07		
	except ribosome	893				$1.3(10^{-04})$	1.07		
GO_CC	intracellular	1173	17%	$1.8(10^{-12})$	$1.4(10^{-09})$	$1.6(10^{-12})$	1.09	0.93	$5.8(10^{-06})$
	except intracellular organelle part	696				$3.3(10^{-01})$	1.01		
	except ribonucleoprotein complex	1037				$5.3(10^{-02})$	1.03		
	except mitochondrion	993				$3.1(10^{-02})$	1.03		
	except cytosolic part	1126				$9.0(10^{-07})$	1.07		
GO_CC	intracellular part	1129	17%	$4.0(10^{-12})$	$3.1(10^{-09})$	$3.5(10^{-12})$	1.10	0.92	$8.2(10^{-06})$
	except intracellular organelle part	652				$2.6(10^{-01})$	1.02		
	except ribonucleoprotein complex	993				$3.8(10^{-02})$	1.03		
	except mitochondrion	949				$2.1(10^{-02})$	1.04		
	except cytosolic part	1082				$8.1(10^{-07})$	1.07		
GO_CC	intracellular membrane-bound organelle	882	17%	$1.6(10^{-10})$	$1.3(10^{-07})$	$1.4(10^{-10})$	1.14	0.85	$2.9(10^{-09})$
	except mitochondrion	702				$1.2(10^{-02})$	1.06		
	except nucleolus	830				$3.5(10^{-06})$	1.10		
	except nuclear speck	859				$9.7(10^{-09})$	1.13		
GO_CC	membrane-bound organelle	882	17%	$1.8(10^{-10})$	$1.4(10^{-07})$	$1.5(10^{-10})$	1.14	0.85	$2.6(10^{-09})$
	except mitochondrion	702				$1.3(10^{-02})$	1.06		
	except nucleolus	830				$3.8(10^{-06})$	1.10		
	except nuclear speck	859				$1.1(10^{-08})$	1.13		
GO_CC	organelle part	479	19%	$4.1(10^{-09})$	$3.2(10^{-06})$	$3.1(10^{-09})$	1.24	0.84	$1.9(10^{-04})$

	except intracellular organelle part	2				$4.1(10^{-01})$	1.46		
	except organelle lumen	280				$2.6(10^{-01})$	1.03		
GO_CC	mitochondrial lumen	51	34%	$1.5(10^{-08})$	$1.2(10^{-05})$	$5.2(10^{-09})$	2.25	0.39	$1.8(10^{-03})$
	except mitochondrial matrix	0				1.0	1.00		
GO_CC	cytoplasmic part	492	19%	$2.2(10^{-08})$	$1.8(10^{-05})$	$1.8(10^{-08})$	1.22	0.99	$4.1(10^{-01})$
	except mitochondrion	312				$9.3(10^{-02})$	1.07		
	except ribosome	406				$2.6(10^{-02})$	1.08		
	except cytosolic part	445				$8.6(10^{-05})$	1.15		
GO_CC	nuclear lumen	130	24%	$3.6(10^{-08})$	$2.8(10^{-05})$	$2.0(10^{-08})$	1.58	0.55	$1.5(10^{-05})$
	except nucleolus	78				$1.1(10^{-02})$	1.28		
	except nuclear speck	107				$3.8(10^{-06})$	1.51		
GO_CC	macromolecular complex	323	20%	$1.0(10^{-07})$	$7.9(10^{-05})$	$7.4(10^{-08})$	1.29	0.76	$2.5(10^{-05})$
	except ribonucleoprotein complex	187				$5.8(10^{-01})$	0.99		
GO_CC	intracellular non-membrane-bound organelle	237	19%	$1.2(10^{-05})$	$9.5(10^{-03})$	$8.8(10^{-06})$	1.28	1.04	$7.2(10^{-01})$
	except ribosome	151				$7.1(10^{-01})$	0.96		
	except nucleolus	185				$3.1(10^{-02})$	1.13		
GO_CC	non-membrane-bound organelle	237	19%	$1.2(10^{-05})$	$9.5(10^{-03})$	$8.8(10^{-06})$	1.28	1.04	$7.2(10^{-01})$
	except ribosome	151				$7.1(10^{-01})$	0.96		
	except nucleolus	185				$3.1(10^{-02})$	1.13		
GO_CC	nucleus	568	17%	$1.7(10^{-05})$	$1.4(10^{-02})$	$1.5(10^{-05})$	1.14	0.78	$1.9(10^{-09})$
	except nucleolus	516				$9.0(10^{-03})$	1.08		
	except nuclear speck	545				$2.4(10^{-04})$	1.12		
GO_CC	nuclear part	175	20%	$3.7(10^{-05})$	$2.9(10^{-02})$	$2.6(10^{-05})$	1.32	0.67	$4.4(10^{-05})$
	except nucleolus	123				$1.1(10^{-01})$	1.11		
	except nuclear speck	152				$1.0(10^{-03})$	1.26		
GO_CC	organellar small ribosomal subunit	11	65%	$4.5(10^{-05})$	$3.4(10^{-02})$	$5.0(10^{-06})$	4.25	0.00	$1.1(10^{-01})$
	except mitochondrial small ribosomal subunit	0				1.0	1.00		
GO_CC	mitochondrial ribosome	20	41%	$4.9(10^{-05})$	$3.8(10^{-02})$	$1.3(10^{-05})$	2.68	0.17	$1.4(10^{-02})$
	except mitochondrial small ribosomal subunit	9				$4.4(10^{-02})$	1.85		
GO_CC	organellar ribosome	20	41%	$4.9(10^{-05})$	$3.8(10^{-02})$	$1.3(10^{-05})$	2.68	0.17	$1.4(10^{-02})$
	except mitochondrial small ribosomal subunit	9				$4.4(10^{-02})$	1.85		
GO_CC	cytoplasm	734	17%	$4.3(10^{-04})$	$2.9(10^{-01})$	$3.9(10^{-04})$	1.09	1.04	$9.3(10^{-01})$
	except mitochondrion	554				$7.4(10^{-01})$	0.98		
	except ribosome	648				$4.9(10^{-01})$	1.00		
	except cytosolic part	687				$4.6(10^{-02})$	1.04		
GO_CC	mitochondrial envelope	66	23%	$4.5(10^{-04})$	$3.0(10^{-01})$	$2.7(10^{-04})$	1.51	0.84	$1.8(10^{-01})$
	except mitochondrial inner membrane	19				$1.3(10^{-02})$	1.69		
GO_CC	mitochondrial membrane	63	23%	$6.9(10^{-04})$	$4.2(10^{-01})$	$4.1(10^{-04})$	1.51	0.82	$1.4(10^{-01})$
	except mitochondrial inner membrane	16				$2.1(10^{-02})$	1.70		
GO_CC	nuclear body	28	29%	$1.1(10^{-03})$	$5.9(10^{-01})$	$5.0(10^{-04})$	1.88	0.25	$1.6(10^{-03})$
	except nuclear speck	5				$2.7(10^{-01})$	1.43		
GO_CC	nucleoplasm	84	21%	$1.2(10^{-03})$	$6.1(10^{-01})$	$7.6(10^{-04})$	1.39	0.46	$6.6(10^{-06})$
	except nuclear speck	61				$3.7(10^{-02})$	1.25		
GO_CC	cytosol	84	20%	$3.7(10^{-03})$	$9.5(10^{-01})$	$2.5(10^{-03})$	1.34	0.93	$3.1(10^{-01})$
	except cytosolic part	37				$9.2(10^{-01})$	0.83		
GO_CC	envelope	87	20%	$5.1(10^{-03})$	$9.8(10^{-01})$	$3.5(10^{-03})$	1.31	0.92	$2.8(10^{-01})$
	except mitochondrial inner membrane	40				$1.4(10^{-01})$	1.18		
GO_CC	organelle envelope	87	20%	$5.1(10^{-03})$	$9.8(10^{-01})$	$3.5(10^{-03})$	1.31	0.92	$2.8(10^{-01})$
	except mitochondrial inner membrane	40				$1.4(10^{-01})$	1.18		
GO_CC	nucleoplasm part	70	21%	$6.1(10^{-03})$	$9.9(10^{-01})$	$4.1(10^{-03})$	1.35	0.49	$8.5(10^{-05})$
	except nuclear speck	47				$1.4(10^{-01})$	1.17		
GO_CC	cell part	1376	15%	$9.7(10^{-03})$	$1.0(10^{-00})$	$9.6(10^{-03})$	1.01	1.00	$2.5(10^{-01})$
	except ribonucleoprotein complex	1240				1.0	0.95		
	except mitochondrion	1196				1.0	0.96		
	except intracellular organelle part	899				1.0	0.92		
	except cytosolic part	1329				$9.9(10^{-01})$	0.99		
GO_CC	cell	1376	15%	$9.7(10^{-03})$	$1.0(10^{-00})$	$9.6(10^{-03})$	1.01	1.00	$2.5(10^{-01})$
	except ribonucleoprotein complex	1240				1.0	0.95		
	except mitochondrion	1196				1.0	0.96		
	except intracellular organelle part	899				1.0	0.92		
	except cytosolic part	1329				$9.9(10^{-01})$	0.99		
GO_MF	structural constituent of ribosome	78	46%	$9.4(10^{-22})$	$2.4(10^{-18})$	$2.4(10^{-22})$	3.08	0.10	$1.2(10^{-07})$
GO_MF	nucleic acid binding	481	21%	$2.4(10^{-19})$	$6.0(10^{-16})$	$3.6(10^{-19})$	1.40	0.64	$1.1(10^{-14})$
	except RNA binding	314				$3.1(10^{-05})$	1.21		
	except translation factor activity, nucleic acid binding	455				$7.0(10^{-17})$	1.38		
	except DNA binding	212				$3.3(10^{-20})$	1.81		
GO_MF	RNA binding	167	29%	$2.6(10^{-19})$	$6.7(10^{-16})$	$1.9(10^{-19})$	1.95	0.34	$1.3(10^{-11})$
	except rRNA binding	159				$1.1(10^{-17})$	1.91		
GO_MF	methyltransferase activity	41	32%	$3.0(10^{-06})$	$7.6(10^{-03})$	$1.1(10^{-06})$	2.11	0.33	$1.3(10^{-03})$
	except S-adenosylmethionine-dependent methyltransferase activity	22				$1.0(10^{-03})$	1.97		
	except RNA methyltransferase activity	31				$5.5(10^{-04})$	1.81		
GO_MF	oxidoreductase activity, acting on the CH-OH group of donors, NAD or NADP as acceptor	26	39%	$5.2(10^{-06})$	$1.3(10^{-02})$	$1.4(10^{-06})$	2.60	0.76	$3.1(10^{-01})$
GO_MF	RNA methyltransferase activity	10	67%	$7.5(10^{-05})$	$1.7(10^{-01})$	$7.7(10^{-06})$	4.47	0.00	$1.5(10^{-01})$
GO_MF	S-adenosylmethionine-dependent methyltransferase activity	19	35%	$6.9(10^{-04})$	$8.3(10^{-01})$	$2.3(10^{-04})$	2.32	0.31	$3.4(10^{-02})$

GO_MF	NAD binding	13	43%	$7.3(10^{-04})$	$8.4(10^{-01})$	$1.7(10^{-04})$	2.91	1.13	$7.2(10^{-01})$
GO_MF	DNA binding	269	18%	$9.2(10^{-04})$	$9.0(10^{-01})$	$7.0(10^{-04})$	1.18	0.74	$1.5(10^{-05})$
GO_MF	nucleotidyltransferase activity	27	27%	$2.6(10^{-03})$	$1.0(10^{-00})$	$1.2(10^{-03})$	1.81	0.17	$3.4(10^{-04})$
GO_MF	unfolded protein binding	25	27%	$3.0(10^{-03})$	$1.0(10^{-00})$	$1.4(10^{-03})$	1.84	0.46	$3.4(10^{-02})$
GO_MF	aminoacyl-tRNA ligase activity	16	33%	$3.9(10^{-03})$	$1.0(10^{-00})$	$1.4(10^{-03})$	2.19	0.52	$1.5(10^{-01})$
GO_MF	ATP-dependent helicase activity	23	28%	$4.1(10^{-03})$	$1.0(10^{-00})$	$1.8(10^{-03})$	1.86	0.20	$2.1(10^{-03})$
GO_MF	translation factor activity, nucleic acid binding	26	26%	$4.7(10^{-03})$	$1.0(10^{-00})$	$2.2(10^{-03})$	1.76	0.85	$3.7(10^{-01})$
GO_MF	exoribonuclease activity, producing 5'-phosphomonoesters	8	50%	$5.3(10^{-03})$	$1.0(10^{-00})$	$1.0(10^{-03})$	3.35	0.53	$4.2(10^{-01})$
GO_MF	rRNA binding	8	47%	$7.9(10^{-03})$	$1.0(10^{-00})$	$1.7(10^{-03})$	3.16	0.00	$1.2(10^{-01})$
GO_MF	structural molecule activity	102	24%	$1.1(10^{-06})$	$2.8(10^{-03})$	$6.1(10^{-07})$	1.59	0.75	$2.5(10^{-02})$
	except structural constituent of ribosome	24				1.0	0.62		
GO_MF	transferase activity, transferring one-carbon groups	41	31%	$3.7(10^{-06})$	$9.4(10^{-03})$	$1.4(10^{-06})$	2.10	0.32	$1.2(10^{-03})$
	except methyltransferase activity	0				1.0	0.00		
GO_MF	oxidoreductase activity, acting on CH-OH group of donors	26	36%	$2.2(10^{-05})$	$5.5(10^{-02})$	$6.9(10^{-06})$	2.42	0.70	$2.4(10^{-01})$
	except oxidoreductase activity, acting on the CH-OH group of donors, NAD or NADP as acceptor	0				1.0	0.00		
GO_MF	helicase activity	35	28%	$2.1(10^{-04})$	$4.2(10^{-01})$	$9.3(10^{-05})$	1.89	0.27	$6.2(10^{-04})$
	except ATP-dependent helicase activity	12				$1.4(10^{-02})$	1.96		
GO_MF	translation regulator activity	30	27%	$1.9(10^{-03})$	$9.9(10^{-01})$	$8.8(10^{-04})$	1.78	0.82	$3.0(10^{-01})$
	except translation factor activity, nucleic acid binding	4				$1.4(10^{-01})$	1.92		
GO_MF	catalytic activity	594	16%	$2.4(10^{-03})$	$1.0(10^{-00})$	$2.1(10^{-03})$	1.09	0.91	$6.7(10^{-03})$
	except methyltransferase activity	553				$5.5(10^{-02})$	1.05		
	except oxidoreductase activity, acting on the CH-OH group of donors, NAD or NADP as acceptor	568				$2.7(10^{-02})$	1.06		
	except nucleotidyltransferase activity	567				$1.6(10^{-02})$	1.07		
	except ATP-dependent helicase activity	571				$1.3(10^{-02})$	1.07		
	except aminoacyl-tRNA ligase activity	578				$9.4(10^{-03})$	1.07		
	except exoribonuclease activity, producing 5'-phosphomonoesters	586				$5.7(10^{-03})$	1.08		
GO_MF	ligase activity, forming aminoacyl-tRNA and related compounds	16	33%	$3.9(10^{-03})$	$1.0(10^{-00})$	$1.4(10^{-03})$	2.19	0.52	$1.5(10^{-01})$
	except aminoacyl-tRNA ligase activity	0				1.0	1.00		
GO_MF	ligase activity, forming carbon-oxygen bonds	16	33%	$3.9(10^{-03})$	$1.0(10^{-00})$	$1.4(10^{-03})$	2.19	0.52	$1.5(10^{-01})$
	except aminoacyl-tRNA ligase activity	0				1.0	1.00		
GO_MF	coenzyme binding	28	25%	$5.1(10^{-03})$	$1.0(10^{-00})$	$2.5(10^{-03})$	1.71	0.77	$2.3(10^{-01})$
	except NAD binding	15				$2.1(10^{-01})$	1.26		
GO_MF	exoribonuclease activity	8	50%	$5.3(10^{-03})$	$1.0(10^{-00})$	$1.0(10^{-03})$	3.35	0.53	$4.2(10^{-01})$
	except exoribonuclease activity, producing 5'-phosphomonoesters	0				1.0	1.00		
GO_MF	ATPase activity	53	21%	$8.6(10^{-03})$	$1.0(10^{-00})$	$5.5(10^{-03})$	1.40	0.70	$4.4(10^{-02})$
	except ATP-dependent helicase activity	30				$1.8(10^{-01})$	1.18		
KEGG	Ribosome	42	63%	$1.1(10^{-17})$	$2.2(10^{-15})$	$1.4(10^{-18})$	3.97	0.00	$3.4(10^{-05})$
KEGG	Purine metabolism	32	28%	$1.2(10^{-03})$	$2.1(10^{-01})$	$5.6(10^{-04})$	1.76	0.31	$5.4(10^{-04})$
KEGG	Aminoacyl-tRNA biosynthesis	14	40%	$1.6(10^{-03})$	$2.7(10^{-01})$	$4.6(10^{-04})$	2.53	0.20	$3.3(10^{-02})$
KEGG	Glycine, serine and threonine metabolism	11	44%	$3.0(10^{-03})$	$4.5(10^{-01})$	$7.3(10^{-04})$	2.79	0.28	$1.1(10^{-01})$
KEGG	Alanine and aspartate metabolism	10	42%	$7.9(10^{-03})$	$7.9(10^{-01})$	$2.1(10^{-03})$	2.64	0.59	$3.2(10^{-01})$

Overall set of over-represented terms (PValue < 0.01; 5th column) are listed here. Every term is followed by the complementary terms of final descendants or ones inbetween which are enriched even excluding genes in descendants. The over-represented terms for themselves are listed first. [a]The proportion of genes within group in total 13,232 genes which were annotated with the specific term [b]EASE score [c]Hypergeometric test for overrepresentation [d]Fold enrichment of the term in the gene group [e]Fold enrichment of the term in the opposite gene group (Table S4, genes expression of which increases as age) [f]Hypergeometric test for underrepresentation of genes in the opposite group
