# Supplementary material for: Transcriptome-Wide Assessment of Human Brain and Lymphocyte Senescence
Source: PLoS One. 2008 Aug 20;3(8):e3024. doi: 10.1371/journal.pone.0003024 (PMC2515343; doi:10.1371/journal.pone.0003024)
Supplement: Table S4 — Terms in the Gene Ontology and KEGG pathway databases enriched among genes that increased expression with advancing age in the human lymphocytes (1430 genes in total 13,232) (0.12 MB PDF) [file pone.0003024.s005.pdf]

Table S4. Terms in the Gene Ontology and KEGG pathway databases enriched among genes that increased expression with advancing age in the human lymphocytes (1430 genes in total 13,232)

Category	Term	Count	%Hit[a]	PValue[b]	Bonferroni	HGeom[c]	Fold[d]	Fold-U[e]	HGe-U[f]
GO_BP	signal transduction	329	16%	$2.1(10^{-10})$	$1.0(10^{-06})$	$1.5(10^{-10})$	1.34	0.72	$8.8(10^{-11})$
	except small GTPase mediated signal transduction	261				$2.7(10^{-06})$	1.28		
	except regulation of signal transduction	255				$2.4(10^{-06})$	1.28		
	except protein kinase cascade	276				$5.4(10^{-08})$	1.32		
	except integrin-mediated signaling pathway	317				$4.6(10^{-09})$	1.31		
GO_BP	immune response	99	22%	$9.2(10^{-10})$	$4.3(10^{-06})$	$4.4(10^{-10})$	1.84	1.02	$6.0(10^{-01})$
	except innate immune response	81				$1.4(10^{-07})$	1.76		
	except lymphocyte mediated immunity	84				$1.0(10^{-07})$	1.76		
	except regulation of immune effector process	92				$1.8(10^{-08})$	1.77		
	except complement activation	92				$1.4(10^{-08})$	1.77		
GO_BP	defense response	82	22%	$7.0(10^{-09})$	$3.3(10^{-05})$	$3.2(10^{-09})$	1.90	0.76	$2.7(10^{-02})$
	except inflammatory response	41				$2.5(10^{-06})$	2.09		
	except cellular defense response	65				$1.8(10^{-06})$	1.78		
	except innate immune response	64				$1.1(10^{-06})$	1.81		
GO_BP	response to external stimulus	87	22%	$1.3(10^{-08})$	$6.2(10^{-05})$	$6.3(10^{-09})$	1.84	0.66	$1.4(10^{-03})$
	except response to wounding	28				$4.8(10^{-04})$	1.91		
	except chemotaxis	60				$1.7(10^{-05})$	1.71		
GO_BP	cytoskeleton organization and biogenesis	74	21%	$6.8(10^{-07})$	$3.2(10^{-03})$	$3.4(10^{-07})$	1.78	0.58	$2.4(10^{-04})$
	except actin cytoskeleton organization and biogenesis	41				$3.2(10^{-04})$	1.72		
GO_BP	positive regulation of cellular process	127	18%	$9.6(10^{-07})$	$4.5(10^{-03})$	$6.0(10^{-07})$	1.51	0.97	$4.1(10^{-01})$
	except positive regulation of cell proliferation	93				$2.8(10^{-04})$	1.41		
	except induction of apoptosis	91				$2.2(10^{-04})$	1.43		
	except positive regulation of signal transduction	101				$3.6(10^{-05})$	1.46		
GO_BP	response to wounding	59	21%	$7.2(10^{-06})$	$3.3(10^{-02})$	$3.5(10^{-06})$	1.80	0.62	$3.0(10^{-03})$
	except inflammatory response	18				$3.6(10^{-03})$	1.95		
	except blood coagulation	41				$1.4(10^{-03})$	1.60		
GO_BP	cell adhesion	79	19%	$7.6(10^{-06})$	$3.5(10^{-02})$	$4.2(10^{-06})$	1.64	0.71	$6.0(10^{-03})$
	except cell-cell adhesion	52				$2.6(10^{-04})$	1.62		
GO_BP	small GTPase mediated signal transduction	68	20%	$2.2(10^{-05})$	$9.9(10^{-02})$	$1.2(10^{-05})$	1.66	0.55	$9.9(10^{-05})$
	except regulation of small GTPase mediated signal transduction	36				$1.4(10^{-03})$	1.66		
GO_BP	regulation of signal transduction	74	19%	$4.9(10^{-05})$	$2.1(10^{-01})$	$2.8(10^{-05})$	1.59	0.79	$3.7(10^{-02})$
	except regulation of small GTPase mediated signal transduction	42				$3.2(10^{-03})$	1.53		
	except positive regulation of signal transduction	48				$1.8(10^{-03})$	1.52		
GO_BP	regulation of biological quality	100	17%	$5.4(10^{-05})$	$2.2(10^{-01})$	$3.4(10^{-05})$	1.47	0.79	$1.5(10^{-02})$
	except blood coagulation	82				$2.8(10^{-03})$	1.34		
GO_BP	regulation of multicellular organismal process	40	23%	$5.5(10^{-05})$	$2.3(10^{-01})$	$2.4(10^{-05})$	1.94	0.95	$4.3(10^{-01})$
GO_BP	cell motility	53	20%	$7.0(10^{-05})$	$2.8(10^{-01})$	$3.6(10^{-05})$	1.73	0.41	$5.7(10^{-06})$
GO_BP	developmental process	288	14%	$8.9(10^{-05})$	$3.4(10^{-01})$	$7.3(10^{-05})$	1.21	0.85	$5.6(10^{-04})$
	except induction of apoptosis	252				$3.5(10^{-03})$	1.16		
	except muscle development	266				$9.5(10^{-04})$	1.18		
	except cytolysis	282				$2.8(10^{-04})$	1.19		
GO_BP	chemotaxis	27	26%	$1.3(10^{-04})$	$4.7(10^{-01})$	$5.1(10^{-05})$	2.20	0.51	$1.7(10^{-02})$
GO_BP	blood coagulation	18	30%	$4.0(10^{-04})$	$8.5(10^{-01})$	$1.2(10^{-04})$	2.54	0.22	$3.6(10^{-03})$
GO_BP	inflammatory response	41	21%	$4.5(10^{-04})$	$8.8(10^{-01})$	$2.3(10^{-04})$	1.74	0.73	$6.1(10^{-02})$
	except complement activation	34				$4.6(10^{-03})$	1.57		
GO_BP	positive regulation of cell proliferation	34	22%	$4.8(10^{-04})$	$9.0(10^{-01})$	$2.3(10^{-04})$	1.86	0.85	$2.6(10^{-01})$
GO_BP	cellular defense response	17	30%	$5.3(10^{-04})$	$9.2(10^{-01})$	$1.6(10^{-04})$	2.57	0.83	$3.7(10^{-01})$
GO_BP	actin cytoskeleton organization and biogenesis	33	22%	$5.7(10^{-04})$	$9.3(10^{-01})$	$2.6(10^{-04})$	1.86	0.62	$2.5(10^{-02})$
GO_BP	induction of apoptosis	36	21%	$8.9(10^{-04})$	$9.8(10^{-01})$	$4.4(10^{-04})$	1.76	0.84	$2.2(10^{-01})$
GO_BP	protein polymerization	14	32%	$1.2(10^{-03})$	$1.0(10^{-00})$	$3.5(10^{-04})$	2.69	0.00	$7.3(10^{-04})$
GO_BP	innate immune response	18	27%	$1.3(10^{-03})$	$1.0(10^{-00})$	$4.6(10^{-04})$	2.31	0.90	$4.5(10^{-01})$
GO_BP	lymphocyte mediated immunity	15	30%	$1.4(10^{-03})$	$1.0(10^{-00})$	$4.5(10^{-04})$	2.54	1.06	$6.6(10^{-01})$
GO_BP	integrin-mediated signaling pathway	12	34%	$1.7(10^{-03})$	$1.0(10^{-00})$	$4.3(10^{-04})$	2.90	0.76	$3.7(10^{-01})$
GO_BP	regulation of immune effector process	7	50%	$3.4(10^{-03})$	$1.0(10^{-00})$	$5.1(10^{-04})$	4.23	1.42	$8.5(10^{-01})$
GO_BP	protein kinase cascade	53	17%	$3.9(10^{-03})$	$1.0(10^{-00})$	$2.4(10^{-03})$	1.47	0.56	$3.2(10^{-04})$
GO_BP	negative regulation of biological process	126	15%	$4.1(10^{-03})$	$1.0(10^{-00})$	$3.2(10^{-03})$	1.25	0.89	$9.3(10^{-02})$
GO_BP	regulation of small GTPase mediated signal transduction	32	20%	$4.2(10^{-03})$	$1.0(10^{-00})$	$2.2(10^{-03})$	1.67	0.74	$8.9(10^{-02})$
GO_BP	cytolysis	6	55%	$5.7(10^{-03})$	$1.0(10^{-00})$	$7.3(10^{-04})$	4.62	0.60	$4.9(10^{-01})$
GO_BP	positive regulation of signal transduction	26	20%	$7.3(10^{-03})$	$1.0(10^{-00})$	$3.7(10^{-03})$	1.72	0.78	$1.7(10^{-01})$
GO_BP	cytoskeleton-dependent intracellular transport	18	23%	$7.4(10^{-03})$	$1.0(10^{-00})$	$3.1(10^{-03})$	1.98	0.60	$8.7(10^{-02})$

GO_BP	complement activation	7	44%	$7.5(10^{-03})$	$1.0(10^{-00})$	$1.4(10^{-03})$	3.70	0.83	$5.6(10^{-01})$
GO_BP	muscle development	22	21%	$8.1(10^{-03})$	$1.0(10^{-00})$	$3.9(10^{-03})$	1.81	1.09	$7.1(10^{-01})$
GO_BP	cell-cell adhesion	27	20%	$8.4(10^{-03})$	$1.0(10^{-00})$	$4.4(10^{-03})$	1.68	0.58	$2.1(10^{-02})$
GO_BP	cell killing	6	50%	$8.8(10^{-03})$	$1.0(10^{-00})$	$1.3(10^{-03})$	4.23	0.55	$4.4(10^{-01})$
GO_BP	cell communication	354	16%	$1.8(10^{-10})$	$8.4(10^{-07})$	$1.3(10^{-10})$	1.32	0.71	$1.1(10^{-12})$
	except signal transduction	25				$3.1(10^{-01})$	1.11		
GO_BP	immune system process	124	20%	$3.9(10^{-10})$	$1.8(10^{-06})$	$2.1(10^{-10})$	1.73	0.97	$4.0(10^{-01})$
	except immune response	25				$5.7(10^{-02})$	1.38		
GO_BP	positive regulation of biological process	139	18%	$4.1(10^{-07})$	$1.9(10^{-03})$	$2.5(10^{-07})$	1.50	1.00	$5.4(10^{-01})$
	except positive regulation of cellular process	12				$1.5(10^{-01})$	1.39		
	except complement activation	132				$3.1(10^{-06})$	1.45		
GO_BP	response to stimulus	241	15%	$1.2(10^{-06})$	$5.6(10^{-03})$	$8.7(10^{-07})$	1.31	0.83	$6.6(10^{-04})$
	except immune response	142				$1.2(10^{-01})$	1.09		
	except response to external stimulus	154				$4.8(10^{-02})$	1.13		
	except defense response	159				$4.3(10^{-02})$	1.13		
GO_BP	intracellular signaling cascade	170	16%	$3.1(10^{-06})$	$1.4(10^{-02})$	$2.1(10^{-06})$	1.39	0.59	$1.5(10^{-10})$
	except small GTPase mediated signal transduction	102				$9.3(10^{-03})$	1.25		
	except protein kinase cascade	117				$2.8(10^{-04})$	1.35		
GO_BP	biological adhesion	79	19%	$7.6(10^{-06})$	$3.5(10^{-02})$	$4.2(10^{-06})$	1.64	0.71	$6.0(10^{-03})$
	except cell adhesion	0				1.0	1.00		
GO_BP	localization of cell	53	20%	$7.0(10^{-05})$	$2.8(10^{-01})$	$3.6(10^{-05})$	1.73	0.41	$5.7(10^{-06})$
	except cell motility	0				1.0	1.00		
GO_BP	behavior	42	22%	$8.7(10^{-05})$	$3.4(10^{-01})$	$4.1(10^{-05})$	1.86	0.49	$7.8(10^{-04})$
	except chemotaxis	15				$8.4(10^{-02})$	1.46		
GO_BP	death	109	17%	$1.1(10^{-04})$	$3.9(10^{-01})$	$7.2(10^{-05})$	1.42	0.90	$1.5(10^{-01})$
	except induction of apoptosis	73				$1.2(10^{-02})$	1.29		
	except cytolysis	103				$5.2(10^{-04})$	1.36		
GO_BP	cell death	109	17%	$1.1(10^{-04})$	$3.9(10^{-01})$	$7.2(10^{-05})$	1.42	0.90	$1.5(10^{-01})$
	except induction of apoptosis	73				$1.2(10^{-02})$	1.29		
	except cytolysis	103				$5.2(10^{-04})$	1.36		
GO_BP	locomotory behavior	32	24%	$1.1(10^{-04})$	$4.1(10^{-01})$	$4.6(10^{-05})$	2.05	0.50	$6.7(10^{-03})$
	except chemotaxis	5				$2.3(10^{-01})$	1.51		
GO_BP	taxis	27	26%	$1.3(10^{-04})$	$4.7(10^{-01})$	$5.1(10^{-05})$	2.20	0.51	$1.7(10^{-02})$
	except chemotaxis	0				1.0	1.00		
GO_BP	actin filament-based process	36	23%	$1.5(10^{-04})$	$5.0(10^{-01})$	$6.6(10^{-05})$	1.93	0.67	$4.4(10^{-02})$
	except actin cytoskeleton organization and biogenesis	3				$5.8(10^{-02})$	3.18		
GO_BP	regulation of body fluid levels	22	29%	$1.6(10^{-04})$	$5.2(10^{-01})$	$5.2(10^{-05})$	2.42	0.34	$6.1(10^{-03})$
	except blood coagulation	4				$1.3(10^{-01})$	1.99		
GO_BP	coagulation	19	31%	$1.9(10^{-04})$	$6.0(10^{-01})$	$5.9(10^{-05})$	2.59	0.21	$2.7(10^{-03})$
	except blood coagulation	1				$2.2(10^{-01})$	4.23		
GO_BP	apoptosis	103	17%	$2.6(10^{-04})$	$7.0(10^{-01})$	$1.8(10^{-04})$	1.40	0.90	$1.6(10^{-01})$
	except induction of apoptosis	67				$2.5(10^{-02})$	1.26		
GO_BP	hemostasis	19	30%	$3.0(10^{-04})$	$7.6(10^{-01})$	$9.5(10^{-05})$	2.51	0.31	$8.5(10^{-03})$
	except blood coagulation	1				$4.0(10^{-01})$	2.12		
GO_BP	programmed cell death	103	16%	$3.1(10^{-04})$	$7.7(10^{-01})$	$2.1(10^{-04})$	1.39	0.90	$1.5(10^{-01})$
	except induction of apoptosis	67				$2.9(10^{-02})$	1.25		
GO_BP	immune effector process	22	27%	$4.1(10^{-04})$	$8.5(10^{-01})$	$1.5(10^{-04})$	2.27	0.81	$2.9(10^{-01})$
	except lymphocyte mediated immunity	7				$7.6(10^{-02})$	1.85		
	except regulation of immune effector process	15				$1.2(10^{-02})$	1.87		
	except complement activation	15				$8.8(10^{-03})$	1.92		
GO_BP	cell proliferation	89	17%	$5.0(10^{-04})$	$9.1(10^{-01})$	$3.3(10^{-04})$	1.42	0.95	$3.2(10^{-01})$
	except positive regulation of cell proliferation	55				$5.5(10^{-02})$	1.24		
GO_BP	positive regulation of programmed cell death	42	20%	$5.3(10^{-04})$	$9.2(10^{-01})$	$2.7(10^{-04})$	1.72	0.90	$3.0(10^{-01})$
	except induction of apoptosis	6				$2.1(10^{-01})$	1.49		
GO_BP	positive regulation of apoptosis	42	20%	$5.3(10^{-04})$	$9.2(10^{-01})$	$2.7(10^{-04})$	1.72	0.90	$3.0(10^{-01})$
	except induction of apoptosis	6				$2.1(10^{-01})$	1.49		
GO_BP	regulation of apoptosis	74	17%	$6.7(10^{-04})$	$9.6(10^{-01})$	$4.2(10^{-04})$	1.46	0.94	$3.3(10^{-01})$
	except induction of apoptosis	38				$8.0(10^{-02})$	1.26		
GO_BP	cell development	134	15%	$7.4(10^{-04})$	$9.7(10^{-01})$	$5.6(10^{-04})$	1.30	0.83	$1.2(10^{-02})$
	except induction of apoptosis	98				$3.7(10^{-02})$	1.19		
	except cytolysis	128				$2.7(10^{-03})$	1.26		
GO_BP	regulation of programmed cell death	74	17%	$7.6(10^{-04})$	$9.7(10^{-01})$	$4.9(10^{-04})$	1.45	0.94	$3.1(10^{-01})$
	except induction of apoptosis	38				$8.7(10^{-02})$	1.25		
GO_BP	induction of programmed cell death	36	21%	$8.9(10^{-04})$	$9.8(10^{-01})$	$4.4(10^{-04})$	1.76	0.84	$2.2(10^{-01})$
	except induction of apoptosis	0				1.0	1.00		
GO_BP	cell surface receptor linked signal transduction	127	15%	$9.0(10^{-04})$	$9.9(10^{-01})$	$6.7(10^{-04})$	1.31	0.73	$2.5(10^{-04})$
	except integrin-mediated signaling pathway	115				$8.0(10^{-03})$	1.23		
GO_BP	acute inflammatory response	13	34%	$9.9(10^{-04})$	$9.9(10^{-01})$	$2.6(10^{-04})$	2.90	0.87	$4.8(10^{-01})$
	except complement activation	6				$3.8(10^{-02})$	2.31		
GO_BP	localization	270	14%	$1.1(10^{-03})$	$1.0(10^{-00})$	$9.2(10^{-04})$	1.17	0.78	$7.5(10^{-07})$
	except cell motility	217				$7.4(10^{-02})$	1.09		
	except cytoskeleton-dependent intracellular transport	252				$7.0(10^{-03})$	1.14		
GO_BP	wound healing	20	26%	$1.4(10^{-03})$	$1.0(10^{-00})$	$5.5(10^{-04})$	2.17	0.25	$1.5(10^{-03})$
	except blood coagulation	2				$6.5(10^{-01})$	0.94		

GO_BP	cellular developmental process	175	15%	$1.5(10^{-03})$	$1.0(10^{-00})$	$1.1(10^{-03})$	1.23	0.82	$2.2(10^{-03})$
	except induction of apoptosis	139				$4.3(10^{-02})$	1.14		
	except cytolysis	169				$4.3(10^{-03})$	1.20		
GO_BP	cell differentiation	175	15%	$1.5(10^{-03})$	$1.0(10^{-00})$	$1.1(10^{-03})$	1.23	0.82	$2.2(10^{-03})$
	except induction of apoptosis	139				$4.3(10^{-02})$	1.14		
	except cytolysis	169				$4.3(10^{-03})$	1.20		
GO_BP	humoral immune response	14	31%	$1.6(10^{-03})$	$1.0(10^{-00})$	$4.6(10^{-04})$	2.63	1.32	$8.7(10^{-01})$
	except complement activation	7				$4.8(10^{-02})$	2.04		
GO_BP	leukocyte mediated immunity	16	29%	$1.6(10^{-03})$	$1.0(10^{-00})$	$5.4(10^{-04})$	2.42	1.06	$6.6(10^{-01})$
	except lymphocyte mediated immunity	1				$5.3(10^{-01})$	1.41		
GO_BP	regulation of cell proliferation	59	18%	$1.6(10^{-03})$	$1.0(10^{-00})$	$1.0(10^{-03})$	1.49	0.81	$7.2(10^{-02})$
	except positive regulation of cell proliferation	25				$2.3(10^{-01})$	1.17		
GO_BP	multicellular organismal process	261	14%	$1.7(10^{-03})$	$1.0(10^{-00})$	$1.4(10^{-03})$	1.17	0.79	$7.4(10^{-06})$
	except regulation of multicellular organismal process	221				$6.3(10^{-02})$	1.09		
	except blood coagulation	243				$1.5(10^{-02})$	1.13		
	except muscle development	239				$1.2(10^{-02})$	1.14		
GO_BP	proteolysis	76	16%	$4.3(10^{-03})$	$1.0(10^{-00})$	$2.9(10^{-03})$	1.36	0.74	$6.9(10^{-03})$
	except complement activation	69				$1.8(10^{-02})$	1.28		
GO_BP	biological regulation	441	13%	$5.0(10^{-03})$	$1.0(10^{-00})$	$4.5(10^{-03})$	1.10	0.98	$2.4(10^{-01})$
	except positive regulation of cellular process	314				$6.2(10^{-01})$	0.99		
	except regulation of biological quality	341				$3.2(10^{-01})$	1.02		
	except regulation of signal transduction	367				$2.2(10^{-01})$	1.03		
	except negative regulation of biological process	315				$1.7(10^{-01})$	1.05		
	except regulation of multicellular organismal process	401				$9.3(10^{-02})$	1.05		
	except regulation of immune effector process	434				$1.2(10^{-02})$	1.08		
	except complement activation	434				$1.2(10^{-02})$	1.09		
GO_BP	multicellular organismal development	188	14%	$5.3(10^{-03})$	$1.0(10^{-00})$	$4.1(10^{-03})$	1.19	0.82	$1.6(10^{-03})$
	except muscle development	166				$3.4(10^{-02})$	1.14		
GO_BP	activation of plasma proteins during acute inflammatory response	7	44%	$7.5(10^{-03})$	$1.0(10^{-00})$	$1.4(10^{-03})$	3.70	0.83	$5.6(10^{-01})$
	except complement activation	0				1.0	1.00		
GO_BP	anatomical structure development	175	14%	$8.1(10^{-03})$	$1.0(10^{-00})$	$6.4(10^{-03})$	1.18	0.85	$8.7(10^{-03})$
	except muscle development	153				$5.0(10^{-02})$	1.13		
GO_BP	system development	143	14%	$8.8(10^{-03})$	$1.0(10^{-00})$	$7.1(10^{-03})$	1.21	0.86	$2.3(10^{-02})$
	except muscle development	121				$6.2(10^{-02})$	1.14		
GO_CC	plasma membrane	269	17%	$7.0(10^{-11})$	$5.5(10^{-08})$	$4.6(10^{-11})$	1.42	0.62	$1.0(10^{-13})$
	except integral to plasma membrane	145				$3.2(10^{-05})$	1.36		
GO_CC	membrane part	462	15%	$1.1(10^{-07})$	$8.9(10^{-05})$	$9.1(10^{-08})$	1.21	0.79	$3.5(10^{-10})$
	except integral to plasma membrane	338				$3.8(10^{-03})$	1.13		
GO_CC	cytoskeleton	127	19%	$1.4(10^{-07})$	$1.1(10^{-04})$	$8.1(10^{-08})$	1.56	0.69	$1.5(10^{-04})$
	except actin cytoskeleton	89				$1.7(10^{-04})$	1.44		
	except microtubule associated complex	108				$8.3(10^{-06})$	1.49		
GO_CC	integral to plasma membrane	124	18%	$2.3(10^{-06})$	$1.8(10^{-03})$	$1.4(10^{-06})$	1.49	0.69	$1.0(10^{-04})$
	except integrin complex	116				$2.5(10^{-05})$	1.43		
GO_CC	actin cytoskeleton	38	23%	$8.5(10^{-05})$	$6.5(10^{-02})$	$3.8(10^{-05})$	1.94	0.52	$4.0(10^{-03})$
	except actin filament	31				$1.2(10^{-03})$	1.75		
GO_CC	integrin complex	8	50%	$1.5(10^{-03})$	$7.0(10^{-01})$	$2.3(10^{-04})$	4.15	0.41	$2.8(10^{-01})$
GO_CC	microtubule associated complex	19	26%	$2.1(10^{-03})$	$8.0(10^{-01})$	$7.8(10^{-04})$	2.16	0.63	$1.2(10^{-01})$
GO_CC	ruffle	11	31%	$6.6(10^{-03})$	$9.9(10^{-01})$	$2.0(10^{-03})$	2.61	0.38	$8.2(10^{-02})$
GO_CC	clathrin-coated vesicle	18	24%	$6.8(10^{-03})$	$1.0(10^{-00})$	$2.9(10^{-03})$	1.99	0.61	$9.8(10^{-02})$
GO_CC	melanosome	18	24%	$7.8(10^{-03})$	$1.0(10^{-00})$	$3.3(10^{-03})$	1.97	1.64	$9.9(10^{-01})$
GO_CC	actin filament	7	44%	$8.2(10^{-03})$	$1.0(10^{-00})$	$1.5(10^{-03})$	3.63	0.00	$7.1(10^{-02})$
GO_CC	membrane	582	15%	$6.4(10^{-13})$	$5.0(10^{-10})$	$5.1(10^{-13})$	1.24	0.78	0.0E+00
	except plasma membrane	313				$1.2(10^{-02})$	1.11		
	except membrane part	120				$1.4(10^{-04})$	1.37		
GO_CC	plasma membrane part	173	17%	$2.5(10^{-07})$	$2.0(10^{-04})$	$1.6(10^{-07})$	1.44	0.66	$2.2(10^{-07})$
	except integral to plasma membrane	49				$3.0(10^{-02})$	1.30		
GO_CC	intrinsic to membrane	400	15%	$8.4(10^{-07})$	$6.6(10^{-04})$	$6.9(10^{-07})$	1.21	0.80	$8.9(10^{-08})$
	except integral to plasma membrane	276				$1.4(10^{-02})$	1.12		
GO_CC	integral to membrane	398	15%	$9.7(10^{-07})$	$7.6(10^{-04})$	$7.8(10^{-07})$	1.21	0.81	$1.2(10^{-07})$
	except integral to plasma membrane	274				$1.5(10^{-02})$	1.12		
GO_CC	intrinsic to plasma membrane	125	18%	$2.7(10^{-06})$	$2.2(10^{-03})$	$1.7(10^{-06})$	1.49	0.69	$9.4(10^{-05})$
	except integral to plasma membrane	1				$6.8(10^{-01})$	0.92		
GO_CC	cytoskeletal part	73	18%	$3.8(10^{-04})$	$2.6(10^{-01})$	$2.3(10^{-04})$	1.49	0.76	$1.9(10^{-02})$
	except microtubule associated complex	54				$1.3(10^{-02})$	1.35		
	except actin filament	66				$2.3(10^{-03})$	1.41		
GO_CC	cytoplasmic membrane-bound vesicle	48	20%	$4.9(10^{-04})$	$3.2(10^{-01})$	$2.6(10^{-04})$	1.65	0.90	$2.9(10^{-01})$
	except clathrin-coated vesicle	30				$1.4(10^{-02})$	1.50		
	except melanosome	30				$1.3(10^{-02})$	1.51		
GO_CC	membrane-bound vesicle	48	20%	$6.5(10^{-04})$	$4.0(10^{-01})$	$3.5(10^{-04})$	1.63	0.89	$2.6(10^{-01})$
	except clathrin-coated vesicle	30				$1.8(10^{-02})$	1.47		
	except melanosome	30				$1.7(10^{-02})$	1.48		
GO_CC	cytoplasmic vesicle	54	18%	$1.3(10^{-03})$	$6.4(10^{-01})$	$7.6(10^{-04})$	1.54	0.97	$4.5(10^{-01})$
	except clathrin-coated vesicle	36				$2.8(10^{-02})$	1.38		
	except melanosome	36				$2.6(10^{-02})$	1.38		

GO_CC	vesicle	54	18%	$2.0(10^{-03})$	$8.0(10^{-01})$	$1.2(10^{-03})$	1.51	0.97	$4.5(10^{-01})$
	except clathrin-coated vesicle	36				$4.0(10^{-02})$	1.34		
	except melanosome	36				$3.7(10^{-02})$	1.35		
GO_CC	cell projection	37	19%	$5.5(10^{-03})$	$9.9(10^{-01})$	$3.1(10^{-03})$	1.58	0.67	$2.8(10^{-02})$
	except ruffle	26				$6.7(10^{-02})$	1.35		
GO_CC	pigment granule	18	24%	$7.8(10^{-03})$	$1.0(10^{-00})$	$3.3(10^{-03})$	1.97	1.64	$9.9(10^{-01})$
	except melanosome	0				1.0	1.00		
GO_MF	protein binding	672	14%	$9.6(10^{-10})$	$2.4(10^{-06})$	$8.0(10^{-10})$	1.16	0.93	$3.3(10^{-03})$
	except receptor binding	588				$5.8(10^{-05})$	1.12		
	except actin binding	623				$5.4(10^{-06})$	1.13		
	except identical protein binding	625				$1.8(10^{-07})$	1.15		
GO_MF	actin binding	49	25%	$9.1(10^{-07})$	$2.3(10^{-03})$	$3.7(10^{-07})$	2.07	0.57	$4.4(10^{-03})$
GO_MF	signal transducer activity	192	16%	$1.3(10^{-06})$	$3.2(10^{-03})$	$8.8(10^{-07})$	1.37	0.79	$4.8(10^{-04})$
	except receptor activity	58				$1.6(10^{-04})$	1.60		
GO_MF	GTPase activity	40	26%	$1.4(10^{-06})$	$3.5(10^{-03})$	$5.1(10^{-07})$	2.24	0.62	$2.7(10^{-02})$
GO_MF	receptor binding	84	20%	$1.5(10^{-06})$	$3.7(10^{-03})$	$7.9(10^{-07})$	1.68	0.76	$1.8(10^{-02})$
	except cytokine activity	54				$2.9(10^{-04})$	1.60		
	except G-protein-coupled receptor binding	71				$4.4(10^{-05})$	1.58		
GO_MF	GTP binding	56	21%	$1.5(10^{-05})$	$3.9(10^{-02})$	$7.6(10^{-06})$	1.79	0.86	$2.0(10^{-01})$
GO_MF	calcium ion binding	85	17%	$2.8(10^{-04})$	$5.0(10^{-01})$	$1.8(10^{-04})$	1.46	0.69	$1.5(10^{-03})$
GO_MF	motor activity	22	26%	$8.2(10^{-04})$	$8.8(10^{-01})$	$3.2(10^{-04})$	2.16	0.78	$2.5(10^{-01})$
GO_MF	receptor activity	134	15%	$1.0(10^{-03})$	$9.3(10^{-01})$	$7.6(10^{-04})$	1.29	0.80	$3.7(10^{-03})$
GO_MF	cytokine activity	30	22%	$1.2(10^{-03})$	$9.5(10^{-01})$	$5.5(10^{-04})$	1.85	0.78	$1.7(10^{-01})$
GO_MF	enzyme regulator activity	85	16%	$3.5(10^{-03})$	$1.0(10^{-00})$	$2.5(10^{-03})$	1.34	0.85	$7.5(10^{-02})$
GO_MF	G-protein-coupled receptor binding	13	30%	$4.0(10^{-03})$	$1.0(10^{-00})$	$1.3(10^{-03})$	2.50	0.46	$8.9(10^{-02})$
GO_MF	identical protein binding	47	17%	$8.3(10^{-03})$	$1.0(10^{-00})$	$5.2(10^{-03})$	1.45	0.76	$5.0(10^{-02})$
GO_MF	cytoskeletal protein binding	66	23%	$1.9(10^{-07})$	$4.9(10^{-04})$	$8.8(10^{-08})$	1.92	0.58	$7.5(10^{-04})$
	except actin binding	17				$3.7(10^{-02})$	1.58		
GO_MF	molecular transducer activity	192	16%	$1.3(10^{-06})$	$3.2(10^{-03})$	$8.8(10^{-07})$	1.37	0.79	$4.8(10^{-04})$
	except signal transducer activity	0				1.0	1.00		
GO_MF	guanyl ribonucleotide binding	56	21%	$1.9(10^{-05})$	$4.8(10^{-02})$	$9.6(10^{-06})$	1.78	0.88	$2.4(10^{-01})$
	except GTP binding	0				1.0	0.00		
GO_MF	guanyl nucleotide binding	56	21%	$1.9(10^{-05})$	$4.8(10^{-02})$	$9.6(10^{-06})$	1.78	0.88	$2.4(10^{-01})$
	except GTP binding	0				1.0	0.00		
KEGG	Regulation of actin cytoskeleton	46	34%	$3.1(10^{-09})$	$6.0(10^{-07})$	$9.5(10^{-10})$	2.44	0.52	$6.4(10^{-03})$
KEGG	Natural killer cell mediated cytotoxicity	31	32%	$1.3(10^{-05})$	$2.6(10^{-03})$	$4.6(10^{-06})$	2.25	0.26	$2.3(10^{-04})$
KEGG	Focal adhesion	36	28%	$3.2(10^{-05})$	$6.4(10^{-03})$	$1.3(10^{-05})$	2.02	0.55	$1.2(10^{-02})$
KEGG	Cytokine-cytokine receptor interaction	39	25%	$4.1(10^{-04})$	$7.8(10^{-02})$	$2.0(10^{-04})$	1.74	0.76	$1.0(10^{-01})$
KEGG	Complement and coagulation cascades	14	38%	$9.7(10^{-04})$	$1.8(10^{-01})$	$2.6(10^{-04})$	2.69	0.68	$2.8(10^{-01})$
KEGG	Leukocyte transendothelial migration	21	27%	$4.2(10^{-03})$	$5.7(10^{-01})$	$1.8(10^{-03})$	1.91	0.65	$1.1(10^{-01})$
KEGG	Cell adhesion molecules (CAMs)	21	26%	$6.6(10^{-03})$	$7.3(10^{-01})$	$3.0(10^{-03})$	1.84	0.55	$4.4(10^{-02})$

Overall set of over-represented terms (PValue < 0.01; 5th column) are listed here. Every term is followed by the complementary terms of final descendants or ones inbetween which are enriched even excluding genes in descendants. The over-represented terms for themselves are listed first. [a]The proportion of genes within group in total 13,232 genes which were annotated with the specific term [b]EASE score [c]Hypergeometric test for overrepresentation [d]Fold enrichment of the term in the gene group [e]Fold enrichment of the term in the opposite gene group (Table S3, genes expression of which decreases as age) [f]Hypergeometric test for underrepresentation of genes in the opposite group
